# pH variation in medical implant biofilms: Causes, measurements, and its implications for antibiotic resistance

**DOI:** 10.3389/fmicb.2022.1028560

**Published:** 2022-10-31

**Authors:** Shayesteh Beladi Behbahani, Sachindra D. Kiridena, Uthpala N. Wijayaratna, Cedric Taylor, Jeffrey N. Anker, Tzuen-Rong Jeremy Tzeng

**Affiliations:** ^1^Department of Biological Sciences, Clemson University, Clemson, SC, United States; ^2^Department of Chemistry, Clemson University, Clemson, SC, United States

**Keywords:** implant infection, pH, biofilm, antibiotic resistance, pH measurement, pH targeted treatment

## Abstract

The advent of implanted medical devices has greatly improved the quality of life and increased longevity. However, infection remains a significant risk because bacteria can colonize device surfaces and form biofilms that are resistant to antibiotics and the host’s immune system. Several factors contribute to this resistance, including heterogeneous biochemical and pH microenvironments that can affect bacterial growth and interfere with antibiotic biochemistry; dormant regions in the biofilm with low oxygen, pH, and metabolites; slow bacterial growth and division; and poor antibody penetration through the biofilm, which may also be regions with poor acid product clearance. Measuring pH in biofilms is thus key to understanding their biochemistry and offers potential routes to detect and treat latent infections. This review covers the causes of biofilm pH changes and simulations, general findings of metabolite-dependent pH gradients, methods for measuring pH in biofilms, effects of pH on biofilms, and pH-targeted antimicrobial-based approaches.

## Introduction and motivation

Most people in developed countries will receive one or more implanted devices during their lifetime, including orthopedic, cardiovascular, dental, and catheter implants. These devices have been a triumph of modern medicine and can improve quality of life and extend life expectancy. However, the device surfaces can be colonized by bacteria and can serve as nidi for infection. The initial infection can occur either during surgery or afterward (hematogenous). Infection rates vary among different implant types, patient profiles, and co-morbidities. Risk factors include traumatic injuries (especially if there is debris in the wound and compromised tissue envelopes), immunosuppressed states, diabetes, smoking, advanced age, and revision of previously infected devices (Tande and Patel, 2014; Abad and Haleem, 2018). For elective procedures such as knee and hip replacements, average infection rates are around 1% but climb to 40% in battlefield injuries due to the traumatic and unclean nature of injuries, and average infection rates are ~50% for cardiac assist devices due to patient profile and co-morbidities (Cabell et al., 2004).

Implant-associated infections are especially concerning because bacteria growing in biofilms on the device surface are highly resistant to antibiotics and the host’s immune system. For many devices, surgical irrigation and debridement coupled with antibiotic therapy are often successful at relatively early stages (within 1 week of surgery or 3 weeks of symptoms; McKenna et al., 2009; Antony et al., 2015); however, at later stages, implants often need to be removed to treat the underlying infection with associated surgeries, hospitalizations, and cost (Moriarty et al., 2016). These costs can be staggering; for example, several studies estimate $100,000 in hospital charges per episode of prosthetic joint infection and $390 k over the lifetime of a 65-year-old (Bozic and Ries, 2005; Kurtz et al., 2012; Garfield et al., 2020). Over half of hospital-acquired infections are associated with implanted medical devices (Darouiche, 2004); therefore, methods are urgently needed for the early detection and treatment of implant infections. Since acidic (low pH) biofilm microenvironments are indicative of poorly perfused and dormant regions, and pH also directly affects biofilm and antibiotics biochemistry, this review seeks to explain how pH is measured in biofilms, how it varies, and how that might affect treatment strategies.

### Structure of this review

First, we provide a brief background on biofilms and their persistence (Section “Background on biofilms”); next, we discuss why pH can be lower or higher in some regions of biofilms (Section “Causes of pH variation”); how pH can be measured in biofilms and what the typical findings are (Section “Measurement of pH variation”); different methods used to measure and image local pH near implants (Section “Bacterial infection diagnosis”); the effect of pH on biofilm, host and antibiotic activity (Section “Effect of pH”), and finally, we cover strategies that target low pH regions of biofilms to treat implant infections (Section “pH targeted bacterial infection treatment”).

## Background on biofilms

Biofilms are groups of microorganisms in which cells stick together on a surface; these adherents are attached within a self-produced matrix of extracellular polymeric substances (EPS). These EPSs usually consist of extracellular DNA, proteins, and polysaccharides. The special three-dimensional structure of biofilms provides an environment for the microorganisms to live as a community that can form on different biotic and abiotic surfaces in industrial, hospital, and natural environments (Watnick and Kolter, 2000). Presumably, this structure serves as protection for microorganisms from the environment and host immunity to increase their chances of survival (Darouiche, 2004; Costerton et al., 2005). In addition, the microorganisms inside this structure are often resistant to antimicrobial agents, showing higher antibiotic resistance rates than planktonic bacteria (Flemming et al., 2016). Biofilms can also form in nutrient conditions that do not permit the growth of planktonic cells and when the bacterial growth rate is decreased (Watnick and Kolter, 2000). In this section, we will briefly review factors affecting biofilm formation and its stages (Section “Biofilm formation”) and the relevance of pH on biofilm formation (Section “Biofilm antibiotic resistance and relevance of pH”).

### Biofilm formation

Biofilms can form on many different surfaces, such as living tissues, medical devices, and industrial or natural aquatic systems, and several factors may affect their formation (Donlan, 2002; Donlan and Costerton, 2002; Alotaibi and Bukhari, 2021). The ideal environment for the attachment of microorganisms onto a surface is the solid–liquid interface between that surface and a liquid medium, such as water or blood. The characteristics of the solid surface, such as surface roughness, surface hydrophobicity, and surface-associated structures, can also be important in the attachment process. Surface roughness is one of the surface characteristics that affect the colonization of microorganisms. Rough surfaces have more surface area; therefore, colonization increases when the surface roughness increases. Surface hydrophobicity can also play a role in the initial attachment; many studies showed that microorganisms attach faster to hydrophobic, nonpolar surfaces, such as Teflon and plastics, than hydrophilic surfaces like glass or metals (Donlan, 2002; Nurioglu et al., 2015). These studies’ results could be complex and contradictory because of the other variables that are present in biofilm formation, such as surface proteins, fimbriae, or other microbial surface-associated structures. The liquid medium has characteristics, such as pH, nutrient level, ionic strength, and temperature that could affect the microorganisms’ attachment. Seasonal changes affecting biofilm formation in aqueous systems might be ascribed to differences in temperature. A laboratory study also demonstrated that increased microbial attachment occurs when nutrient concentrations are higher (Otto, 2008). Cell surface features like hydrophobicity, fimbriae, flagella, and EPS production can influence the attachment rate. Most bacteria studied are negatively charged and contain hydrophobic surface components (Donlan, 2002). Different bacterial strains might vary in hydrophobicity. Some studies did not find a relationship between the bacterial surface hydrophobicity and the extent of initial binding to either a hydrophilic or hydrophobic substrate (Cerca et al., 2005).

**Figure 1:**
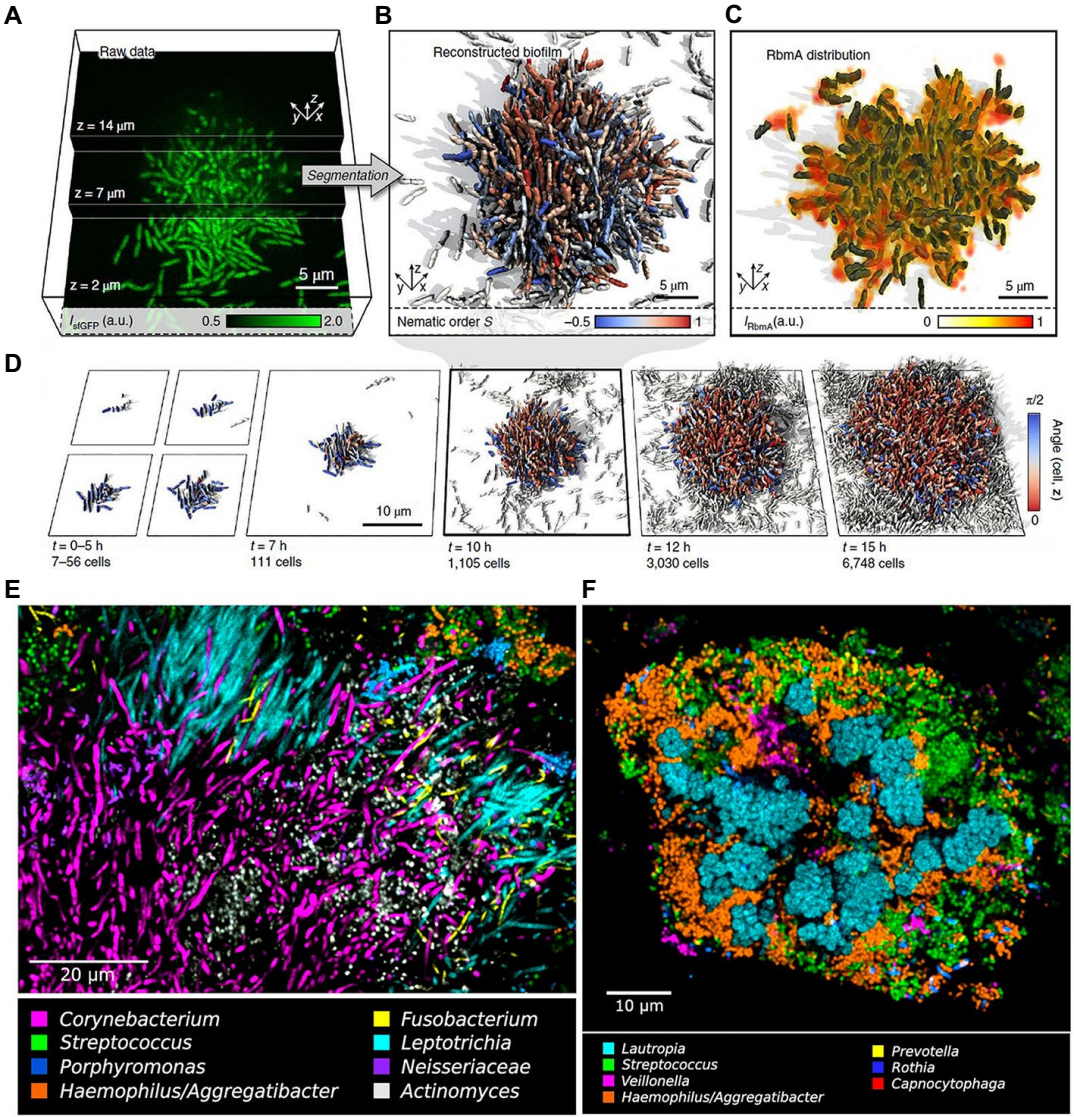
Biofilm growth and structure*.* **(A)** Confocal microscopy images of *Vibrio cholerae* cells constitutively expressing a green fluorescent protein at three different *z* planes. **(B)** 3D reconstruction of the biofilm shown in **(A)**, cells are colored according to the nematic order parameter *S* = <3_2 (n_*i* _ n_*j*) −1_2 > 2 in its vicinity. High-time-resolution (Δ*t* = 5–10 min) imaging allows tracking of cell lineages. White cells are not direct descendants of the biofilm founder cell. **(C)** Shows the extracellular matrix protein RbmA that mediates cell–cell adhesion and is distributed throughout the biofilm. **(D)** Biofilm growth series over time. Starting from initial attachment to irreversible attachment, maturation and dispersion. Cells are colored according to the cellular alignment with the *z* axis (Hartmann et al., 2019). **(E)** Localization of Actinomyces within hedgehogs, in patches within the base region of hedgehogs, and adjacent to them in a dental plaque microbiome. **(F)** Cauliflower structure in plaque composed of Lautropia, Streptococcus, Haemophilus/Aggregatibacter, and Veillonella. Scattered cells of Prevotella, Rothia, and Capnocytophaga are also visible (Welch et al., 2016). These figures were reproduced with permission from Hartmann et al. (2019).

Biofilm development involves several steps: initial attachment of microorganisms, biofilm maturation, and finally, the dispersal of biofilm cells (Donlan and Costerton, 2002; Dunne, 2002; Arciola et al., 2012; Veerachamy et al., 2014). Figure 1 shows how the biofilm structure changes over time *in vitro* from a few bacteria which initially grow exponentially. As shown in Figure 1D, the first step is the initial attachment of microorganisms. On *Staphylococcus aureus,* one of the most prevalent bacteria in implant-associated infection, the microbial surface components recognizing adhesive matrix molecules (MSCRAMMS) are expressed (Heilmann et al., 2003). These MSCRAMMs help the bacteria to attach to different surfaces *via* various mechanisms (Wann et al., 2000). Polysaccharide intercellular adhesin (PIA), with the chemical composition poly-N-acetylglucosamine (PNAG) produced by the *ica* gene locus, is the main molecule responsible for intercellular adhesion (Cramton et al., 1999; Boles et al., 2005). De-acetylation of N-acetylglucosamine in PIA positively charges the molecule. Hence, PIA attaches by electrostatic interaction to the bacterial surface that is normally negatively charged due to its teichoic acid contents (Sadovskaya et al., 2005). When the matrix develops a complex structure, the attachment becomes irreversible (Monroe, 2007). The next step is the maturation phase, known by extracellular aggregation of adhesive proteins and polysaccharides with biofilm structural forces that make three-dimensional mushroom-like cell towers and fluid-filled channels between the towers through which nutrients are delivered to cells deeper in biofilm. The final step is the dispersal phase, which leads to dissemination of the bacteria to new infection sites (Otto, 2008). Single cells or large cell clusters may detach from the biofilm surface area, which is also controlled by the quorum sensing in staphylococci. *In vivo* biofilms are usually poly-colonial and consist of many species with complex structures, as shown in Figures 1E,F of oral biofilms (Welch et al., 2016).

### Biofilm antibiotic resistance and relevance of pH

**Figure 2:**
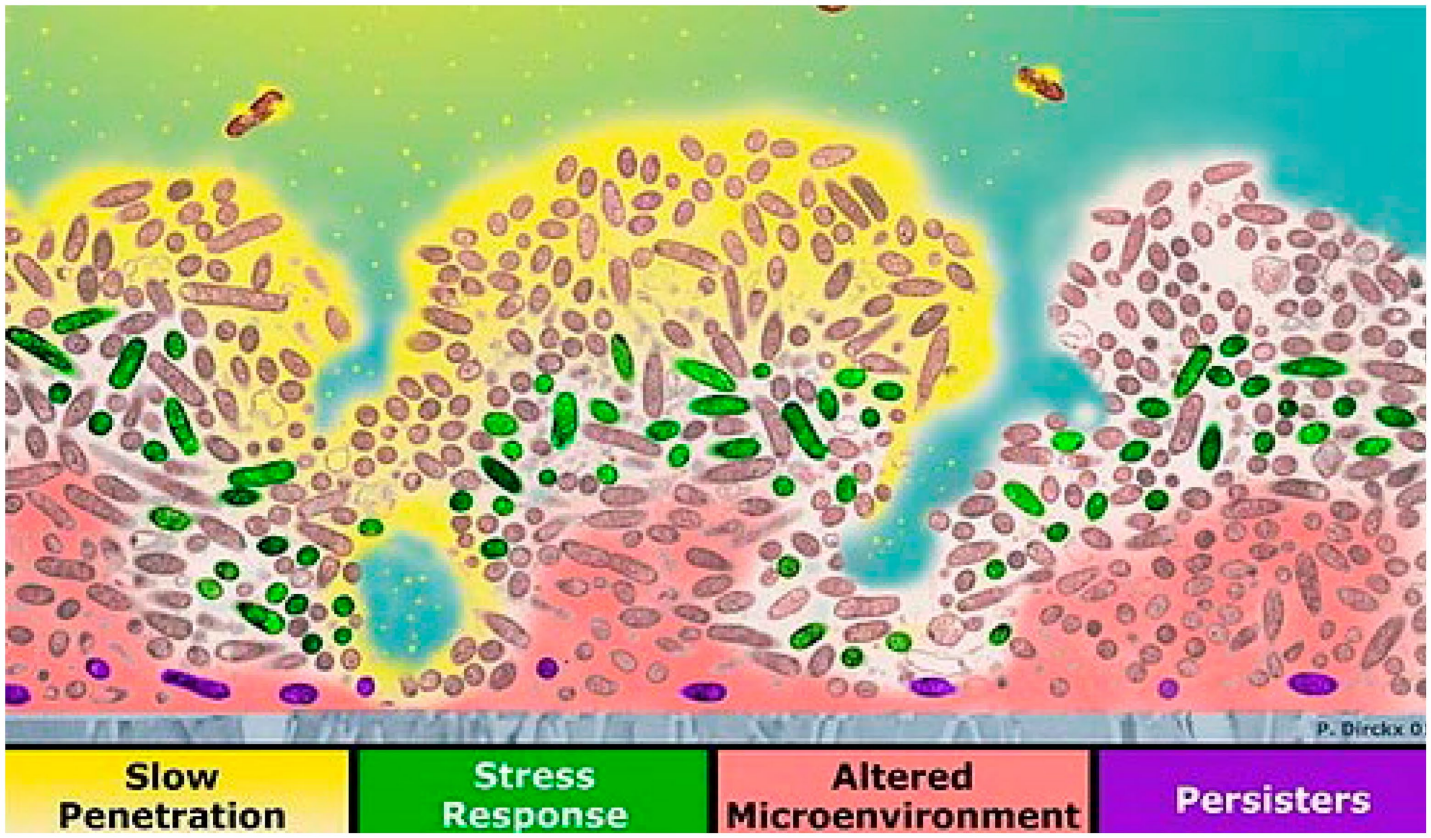
Four mechanisms of reduced susceptibility to antimicrobials in biofilm: slow antibiotic penetration (antibiotics shown in yellow), stress response (e.g., green cells), altered microenvironment (pink), and resistant phenotype [purple persister cells; reproduced with permission from Chambless et al. (2006)].

Four mechanisms of biofilm antibiotic resistance have been described (see Figure 2), and these are either the cause or the effect of pH changes within the biofilm (Stewart, 2002). (1) The first mechanism is poor antimicrobial penetration, in which the antimicrobial agents penetrate slowly or incompletely through the biofilm. Co-metabolism of the antimicrobial agents by the biofilm as it consumes other substrates will decrease the concentration of the antimicrobial agents to a level that would be ineffective in the deeper regions of the biofilm (Stewart, 1996, 2002). Regions that have low perfusion of drugs also show slow penetration of oxygen into the biofilm and slow removal of carbon dioxide and acidic byproducts out of it. Anaerobic respiration, due to low oxygen levels, and poor clearance of metabolic products result in low pH in these regions. (2) With the second mechanism, the altered microenvironment and slow growth enable bacteria in a biofilm to become more resistant to antimicrobial agents. Within a biofilm, there is a micro-gradient found in the concentration of critical metabolic substrates and products that leads to the slow-growing or stationary phases of bacterial cells. Bacteria in the slow-growing phase are less susceptible than bacteria in the growing phase and can survive antibacterial challenges (Donlan, 2002; Stewart, 2002; Gilbert et al., 2003). pH gradients in the biofilm may positively or negatively impact the activity of antibiotics, depending on the type of antibiotic used (Venglarcik et al., 1983; Stewart, 2002). (3) The third mechanism of biofilm protection is stress response defenses induced by biofilm-forming bacteria when they encounter an environmental challenge (Stewart, 2002; Chambless et al., 2006). These stresses alter gene expression patterns and cell physiology resulting in specific and highly regulated adaptive responses (Poole, 2012). These stress responses may impact antimicrobial susceptibility either directly or indirectly. Initiation of stress responses that result in recruitment of resistance determinants (e.g., antimicrobial efflux), development of resistance mutations, changes to antimicrobial targets, alterations to the membrane barrier functions, and promotion of resistant growth modes directly affect antimicrobial activity. Low pH has been found to promote the expression of a gene encoding multidrug resistance efflux pump NorB resulting in reduced susceptibility to moxifloxacin in *S. aureus* (Truong-Bolduc et al., 2011; Poole, 2012). The *rpoS* gene, which encodes the general stress response sigma factor, has been found to respond to a pH downshift from pH 7 to pH 5 in *Escherichia coli* (Hengge-Aronis, 2002). The stress responses may promote physiological changes that indirectly affect antimicrobial activity; for example, stress-induced slow growth rate or dormancy in regions under nutrient stress will impact antimicrobials that target rapidly growing cells (Mah and O’Toole, 2001). Therefore, pH may also be considered to indirectly affect antimicrobial susceptibility, since low pH can be observed in regions under oxidative stress and low nutrients. (4) The fourth mechanism of biofilm antimicrobial resistance is the possibility of a highly protected phenotype of microorganisms in a biofilm. Cells in this state are called persisters (Chambless et al., 2006). There may be a possible link between persisters and stress. Genes responsible for the persister state may include those that code for factors that determine the entry into and exit out of the persister state as well as those affecting stress responses (Stewart, 2002). The *rpoS* gene, which encodes the general stress response factor (also responsive to pH), has been linked to persister formation in *E. coli* and *Pseudomonas aeruginosa*. In response to acidic pH, *E. coli* produced increased levels of ampicillin-resistant persisters compared with wild-type or untreated cells (Hong et al., 2012). It seems likely that a combination of these factors determines the overall protection of the biofilm, and pH indirectly or directly affects the antimicrobial susceptibility of these cells.

Antibiotics can be divided into two groups based on their effect on the growth of cells with the bactericidal antibiotics killing the bacteria and the bacteriostatic antibiotics suppressing the growth of bacteria. The susceptibility of bacteria to an antibiotic could be distinguished as resistant, tolerant, or persistent based on their modes of survival. Antibiotic resistance describes the bacteria with reduced susceptibility to an antibiotic and continues to grow in its presence. The level of antibiotic resistance can by quantified by the minimum inhibitory concentration (MIC), which is defined as the minimum concentration of an antimicrobial required to prevent the growth of cells. Antibiotic tolerance, on the other hand, describes bacteria with an increased minimum duration (MDK) for killing but with little or no change in the MIC than their susceptible counterparts (Handwerger and Tomasz, 1985; Balaban et al., 2019). Whereas, antibiotic persistence describes a subset of non-replicative population with similar MIC but significantly higher MDK for killing enabling them to persist in an environment (Kester and Fortune, 2014; Balaban et al., 2019).

Mechanisms of reduced susceptibility to antimicrobials in biofilm are shown in a cross-section of a biofilm (Figure 2). The attachment surface is shown in gray. The yellow phase contains the antibiotic at the top, where antimicrobial penetration is restricted in the presence of EPSs. In the green areas, some bacteria change their activity in response to antimicrobial stress. The microenvironment in the deeper area is altered to resist eradication (pink). Persister cells are present at a higher ratio deep in the biofilm (violet; Stewart and Costerton, 2001; Chambless et al., 2006). Biofilms provide an excellent environment for transferring extrachromosomal DNA (plasmids) through conjugation because the cells in a biofilm are closer in proximity, and there is a higher chance for cell-to-cell contact. These plasmids may encode for resistance to antimicrobial compounds (Donlan, 2002; Donlan and Costerton, 2002).

In summary, biofilms have multiple stages that can be targeted: initial attachment, irreversible attachment, growth, etc. Once mature, microorganisms in the heterogeneous and persistent/quiescent regions of the biofilm are hard to eradicate. Biofilm resistance develops for multiple reasons, which can correlate with low pH and/or are influenced by pH changes. Therefore, understanding the microenvironment is a key factor in the development of detection and treatment strategies.

## Causes of pH variation

**Figure 3:**
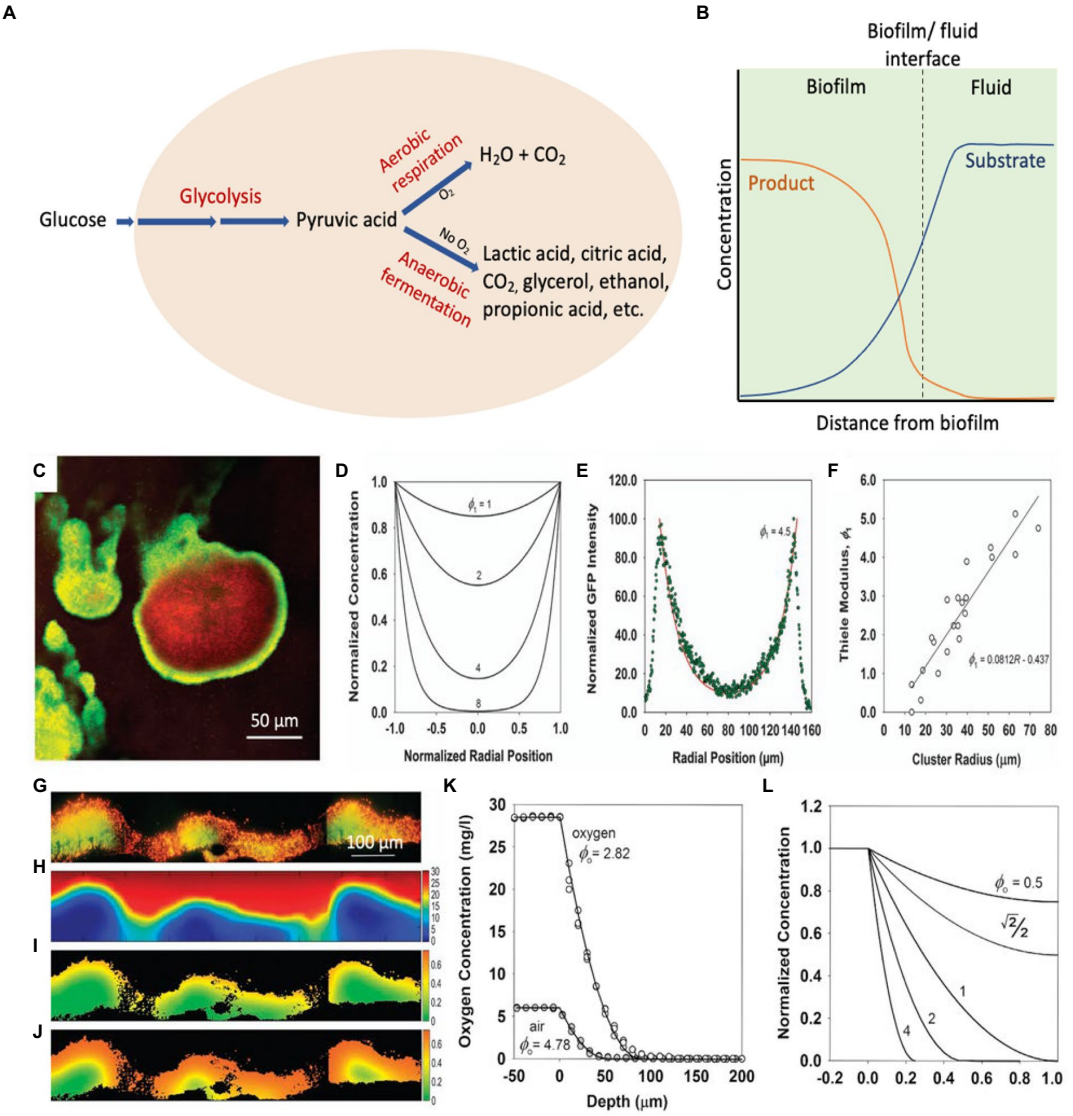
Mechanisms of local acidosis and hypoxia with the comparison between reaction–diffusion simulations and experiments. **(A)** Schematic diagram of cellular glucose metabolic pathways resulting in acidic metabolites. **(B)** Concentrations of the metabolic substrate and metabolic product with distance from the biofilm. **(C)** The pattern of GFP expression (green) and biomass counterstain (red) in a cell cluster demonstrating a growth rate gradient within a biofilm. The scale bar represents 50 μm (Stewart et al., 2016). **(D)** Calculated concentration profiles of a substrate in a hemispherical cluster for varying values of the Thiele modulus, ϕ_1_ under first-order reaction kinetics (Stewart et al., 2016). **(E)** Fit of a theoretical curve (ϕ_1_ = 4.5) fitted to an experimental GFP intensity (Stewart et al., 2016). **(F)** Determination of Thiele modulus by fitting experimental data versus cluster radius (Stewart et al., 2016). **(G)** The relative growth rate of a biofilm using acridine orange-stained frozen section (red/orange represent regions of relatively rapid growth; green/yellow regions represent slow growth). The scale bar represents 100 μm (Wentland et al., 1996; Stewart et al., 2016). **(H)** Simulated glucose concentration in mg L^−1^ (bulk fluid glucose concentration of 30 mg L^−1^; Stewart et al., 2016). **(I)** Simulated growth rate in h^−1^ (bulk fluid glucose concentrations of 10 mg L^−1^; Stewart et al., 2016). **(J)** Simulated growth rate in h^−1^ (bulk fluid glucose concentrations of 25 mg L^−1^; Stewart et al., 2016). **(K)** Experimental oxygen concentration profile in a biofilm (gray circles – exposed to air, open circles – exposed to oxygen-enriched gas, solid lines – theoretical curves fitted to two data sets; Stewart et al., 2016). **(L)** Theoretical concentration profile for a metabolic substrate with varying values of Thiele modulus, ϕ_o,_ under zero-order reaction kinetics (Stewart et al., 2016). These figures were reproduced with permission from Stewart et al. (2016) and Wentland et al. (1996).

A key feature of a biofilm is the development of gradients of various biochemical parameters resulting in a heterogeneous microenvironment. The metabolic activities of microbial cells and diffusional processes result in concentration gradients of metabolic substrates and products within the biofilm (Costerton et al., 1994; Stewart and Franklin, 2008). Generally, due to low oxygen levels, anaerobic fermentation in the biofilm leads to local depletion of nutrients and accumulation of metabolic waste products, such as lactic acid, citric acid, carbon dioxide, propionic acid, glycerol, ethanol, etc., within the biofilm (Figure 3A). Therefore, it is observed that the concentration of substrate/nutrients decreases with an increase in depth of a biofilm and distance from source (Stewart and Franklin, 2008; Figure 3B). In contrast, metabolic product concentrations are found to increase with an increase in depth into the biofilm (Figure 3B; Damgaard et al., 2001). The products are transported from the source, down the concentration gradient, and out of the biofilm. Thus, the production and accumulation of acidic metabolites and waste products from cellular processes will result in higher concentrations of these within the biofilm in comparison to the outside, which may lead to a lowering of the local pH and affect the physiological state of the bacteria (Stewart and Franklin, 2008).

The distribution of solutes in the biofilm is governed by the simultaneous production, consumption, and diffusion of solutes. The reaction–diffusion theory has been widely applied to understand microscale chemical gradients in biofilms, and mathematical models have been developed to simulate these gradients (LaMotta, 1976; Stewart, 2003; Xavier et al., 2005; Petroff et al., 2011; Stewart et al., 2016). Stewart and coworkers used a bacterial strain with an inducible fluorescent protein in order to visualize the growth rate gradient in a biofilm and compared the experimental results with the mathematical simulations (Stewart et al., 2016). The experiment was performed by growing a *P. aeruginosa* biofilm containing isopropylthio-β-D-galactoside-inducible green fluorescent protein (GFP) in the absence of an inducer. After induction of GFP and counter-staining with a red dye, a green color was developed corresponding to the local expression of GFP; the red stain shows the distribution of biomass independent of metabolic activity. As seen in Figure 3C, the green GFP expression pattern is brighter near the periphery of the cluster (showing more GFP expression) and dimmer toward the center (less GFP expression), demonstrating the relative growth rate of bacteria in the different regions of the biofilm. However, it is worth noting that since the induction of GFP gene requires the diffusion of IPTG inducer and the maturation of GFP chromophore could be impacted by oxygen concentration gradient, the data only suggest that organisms located on the peripheral of biofilm exhibited higher metabolic activity. The calculated concentration profiles of a substrate in a hemispherical cluster for several values of the Thiele modulus, ϕ_1_ (Figure 3D), can be compared and fitted with the image analysis of GFP intensity in the biofilm cluster (Figure 3E). The image analysis of multiple biofilm clusters was used to determine the Thiele modulus for each, and Figure 3F shows the linear dependence of the Thiele modulus with the cluster radius, which is as expected, and the slope of the fitted line can be used to estimate the first-order reaction rate coefficient.

In an *in vitro* study by Wentland et al. (1996), the relative growth rate of a *Klebsiella pneumoniae* biofilm (Wentland et al., 1996; Stewart et al., 2016) was compared to the simulated distribution of glucose. The experimental results (Figure 3G) show higher growth rates (orange or red color) on the biofilm-bulk interface and lower growth rates (yellow and green colors) within the biofilm. The variation in the growth rates can be clearly seen in the thicker, denser regions compared to the thinner regions. The simulation of the glucose concentration also shows similar results in that the glucose concentration is diminished within the larger clusters (Figure 3H). Changing the bulk glucose concentration also changed the growth rate patterns, with a low growth rate at low glucose concentrations (Figure 3I), and higher growth rates at high glucose concentrations (Figure 3J). The simulated growth pattern at a bulk glucose concentration of 25 mg L^−1^ fitted with the experimental growth pattern (Figure 3J). The utilization of a fluorescence acridine orange nucleic dye to stain the *K. pneumoniae* biofilm cross sections to determine the growth rate of *K. pneumoniae* in biofilm is oxygen- and inducer-independent, which minimized the potential drawbacks.

Oxygen concentration profiles in biofilms demonstrate a rapid decline in oxygen concentration with increased depth due to respiration by cells in the upper level of the biofilm (Stewart, 2003). The local oxygen concentration depends on the balance between consumption and diffusion of oxygen (Zhang et al., 1995). The experimental oxygen concentration profile of a *P. aeruginosa* biofilm (Figure 3K) fits well with the theoretical concentration profile for a metabolic substrate with zero-order reaction kinetics (Figure 3L), showing low oxygen levels with an increase in depth of the biofilm (Stewart et al., 2016). The low oxygen levels in these regions result in a local acidic microenvironment within the biofilm. Anaerobic fermentation at low oxygen levels causes the production of organic acids, such as lactic acid and acetic acid, and the slow diffusion causes the accumulation of these products. As a result, a local acidic microenvironment is created within the biofilm, and the presence of the extracellular polymer matrix enables the pH to be relatively unaffected by external pH (Fuchs et al., 2007; Radovic-Moreno et al., 2012).

Many studies have measured extracellular pH in biofilms since the variation of pH within a biofilm may have implications on the microbial physiology and biofilm structure (Allan et al., 1999; Vroom et al., 1999; Hou et al., 2017). Hou et al. studied the pH distribution on an anodic biofilm using a pH microelectrode (Hou et al., 2017). As the pH microelectrode tip moved from the bulk solution (~3,500 mm) toward the anode biofilm surface (0 mm), the pH decreased gradually. A study by Siegrist and Gruger demonstrated the effect of mass transfer and pH on ammonium uptake of nitrifying bacteria (Siegrist and Gujer, 1987). The ammonium oxidation rate was observed to decrease with lower pH due to decreasing volume uptake rate, whereas nitrite oxidation increased with lower pH (Siegrist and Gujer, 1987). Similar gradients can also be seen in bacterial quorum sensing molecules, which are intercellular signaling molecules produced by bacteria (Dalwadi and Pearce, 2021). Since low pH regions are indicative of high concentrations of bacteria, it is expected that quorum sensing molecules produced by these bacteria will also be in relatively high concentrations within a biofilm (Dalwadi and Pearce, 2021).

## Measurement of pH variation

The extracellular pH in normal tissue and interstitial fluid around an implant surface is typically around 7.4, but during bacterial infection, the local pH around the implant can drop to 5.5 (Ma et al., 2010). As a result of aerobic respiration and anaerobic fermentation, bacteria produce acidic metabolites, such as lactic acid, acetic acid, formic acid, and carbonic acid (Zhu et al., 2007; Wang et al., 2014). Production of these acidic metabolites generates local acidic and hypoxic regions within a biofilm (Wang et al., 2014). The pH drop around the implant can be an indication of infection (Dong et al., 2017). Inflammatory responses by the human immune system also contribute to local acidosis. Local pH drops due to massive infiltration of neutrophils and macrophages (Pichavant et al., 2012). Acute inflammation caused by surgery can lead to a pH decrease in the local site. The level of pre-inflammatory markers is highest hours after the surgery and will decrease drastically over 24 h depending on the magnitude of the damage (Szelenyi and Urso, 2012).

Biochemical parameters are in a gradient within a biofilm (Costerton et al., 1994; Stewart and Franklin, 2008). Different methods have been used to measure the pH gradient within biofilms, using pH microelectrodes, pH-responsive dyes, and pH-responsive nanoparticles. Each has its own advantages and limitations (see Table 1) and will be described below.

**Table 1:** Summary of the methods used to measure pH gradient in biofilms.

	pH microelectrodes	pH fluorescent dyes	Fluorescent nanoparticles
pH measurement method	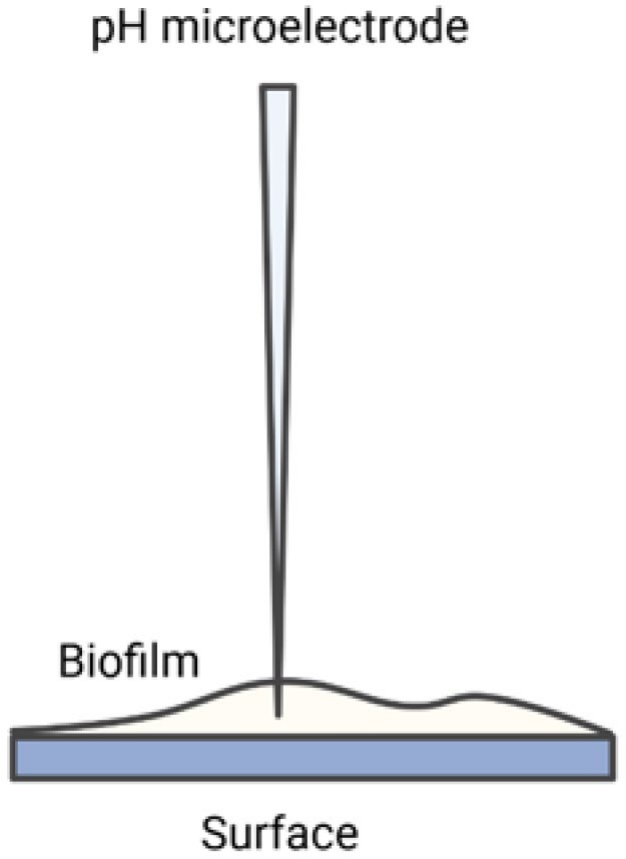	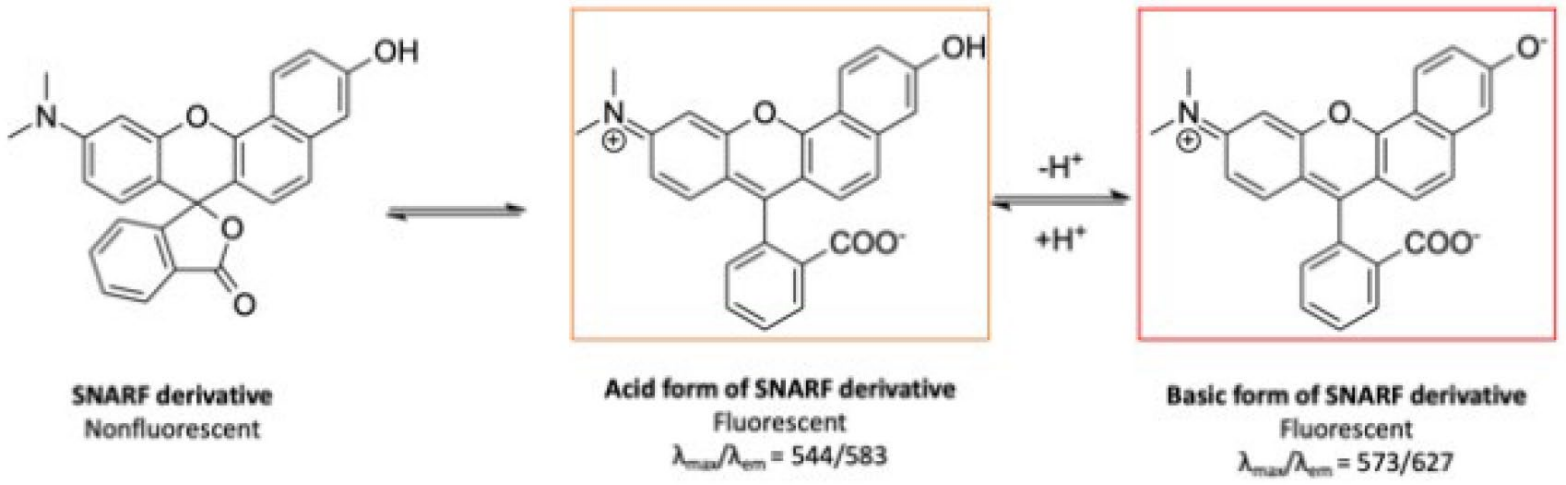 Nakata et al. (2014)	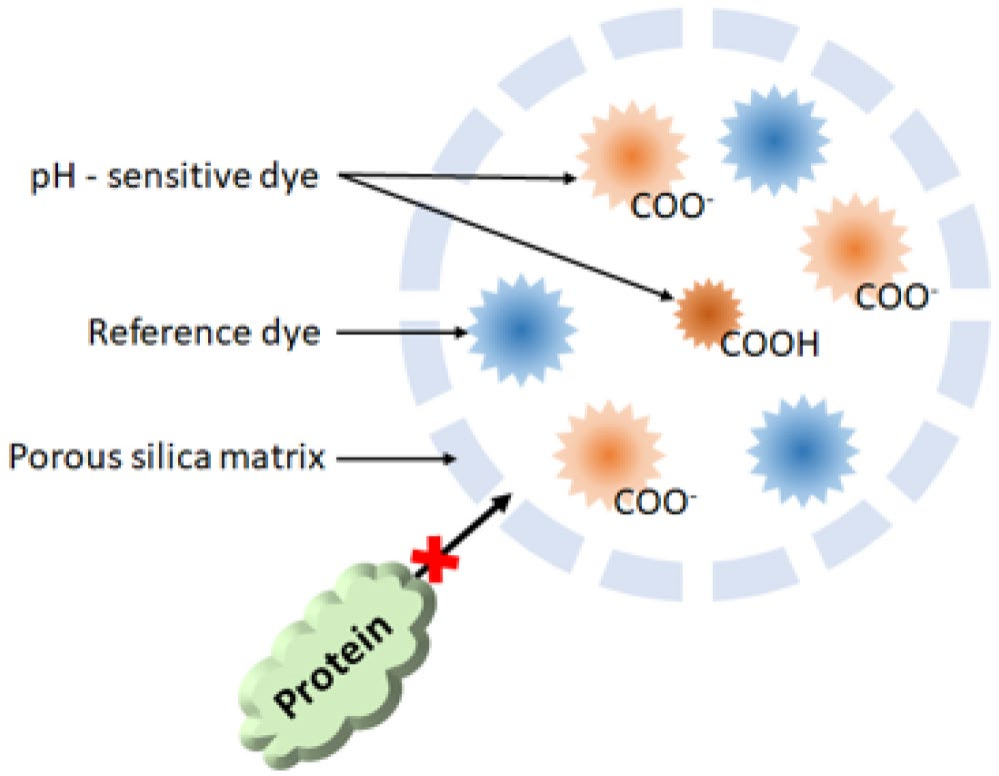
Advantages	High resolution Simple	Imaging of a large region of the biofilm simultaneously Does not damage biofilm structure Does not drift compared to microelectrodes	Able to add reference dyes, additional indicator dyes, and other analytes Ability to change surface characteristics and size for specific targeting Does not damage biofilm structure Reduced interference from proteins
Disadvantages	Fragile pH drifting Biofouling Require frequent calibration Destructive to biofilm Cannot evaluate the horizontal pH profiles in real-time Variable accuracy and precision between different electrodes	Interference due to interactions with biofilm proteins Poor photostability; undergo quenching, photobleaching Limited brightness Require calibration *in situ*	Not typically available commercially Low diffusion rates within biofilm Potential for leaching over long term if not covalently bound Potentially different intracellular and extracellular distribution based on surface chemistry, size, and biofilm Change in calibration graph of the dye due to change in local environment. May also cause the calibration graph to be stretched

### pH microelectrodes

Extracellular pH at a specific location in a biofilm can be measured using pH microelectrodes (Allan et al., 1999; Wang et al., 2013; Xiao et al., 2013). Microelectrodes are electrochemical sensors with a tip diameter of less than 20 μm (Wightman, 2006). pH microelectrodes consist of an ion-permeable membrane that reports the log of the hydronium ion activity (concentrations) as changes in current or potential within the electrode system (reference and sensing electrode; Vonau and Guth, 2006). Among the microelectrodes used to study biofilms, iridium/iridium oxide-based microelectrodes have been found to be effective with respect to rigidity, range, calibration, and reproducibility (VanHoudt et al., 1992). Measuring pH using microelectrodes provides simplicity and high pH resolution over a wide pH range; however, they can be fragile, undergo potential drift and biofouling over time (requiring frequent recalibration), and show variable accuracy and precision between different electrodes. In addition, the technique requires the insertion of the microelectrode into the biofilm itself, which can be destructive to the biofilm and only allows for measuring one position at a time (need to control position with a stage), and it does not allow for evaluation of the horizontal pH profiles in real-time (Vonau and Guth, 2006; Saccomano et al., 2021).

**Figure 4:**
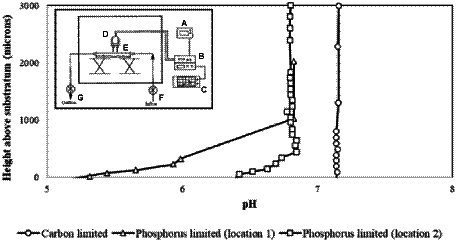
The specimen pH profile of *Citrobacter* biofilm. The circle denotes the specimen pH profile of a *Citrobacter* biofilm pre-grown and measured under carbon limitation; measurements were recorded under laminar flow (9.5 ml/min). The electrode penetrated through the biofilm to the substratum. The square and triangle denote the specimen pH profiles of two locations measured in a *Citrobacter* biofilm pre-grown under carbon limitation and then imaged with a phosphorus limiting medium; laminar flow conditions were the same. The profiles suggest that the diffusion boundary layer is approximately 400 mm in depth. The inset illustrates the overview of the pH microelectrode measurement set-up; (A) power supply, (B) preamplifier, (C) chart recorder, (D) measurement electrode and reference electrode, (E) glass culture flow cell, (F) culture inflow, (G) culture outflow. Data digitized and replotted from Allan et al. (1999).

For example, Allan et al. used pH microelectrodes to measure pH as a function of distance from a surface in *Citrobacter* sp. biofilms with either a carbon-limiting medium and/or a phosphate-limiting medium (Figure 4; Allan et al., 1999). No pH variations were observed in a carbon-limiting medium (constant pH 7.15). By contrast, in the phosphate-limiting medium, the biofilm became more acidic near the surface, dropping as low as pH 5.2 with a maximum gradient of about 1 pH unit/100 μm. This pH drop is attributed to the generation of acidic fermentation products with slow diffusion through the biofilm resulting in a pH gradient (Figure 4; Allan et al., 1999). Although using pH microelectrodes can provide a pH depth profile, the spatial resolution is not very good (relatively coarse), and the “image” is usually one-dimensional.

### Fluorescent pH indicator dyes

Extracellular pH in biofilms can also be imaged using fluorescent pH indicator dyes in combination with a confocal microscopy readout. pH-driven changes in dye protonation results in changes in the fluorescence emission intensity, spectrum, or lifetime (Han and Burgess, 2010; Fulaz et al., 2019). As with any pH indicators, the response of a fluorescent pH dye is a function of the concentration of the acidic and basic forms of the dye, according to the Henderson–Hasselbalch equation (Berezin et al., 2011; Gotor et al., 2017). The largest change in protonated and deprotonated dye concentrations occurs when the pH is equal to the log of the acid dissociation constant of the dye (pK_a_; Berezin et al., 2011) and the dynamic range is usually within +/− 1 pH unit of the pK_a_. Thus, for *in vivo* studies, the indicator dye pK_a_ should be within the physiologically relevant pH range (around 7). For imaging through highly scattering media, it is also helpful to have red or near-infrared excitation and emission (650–900 nm; Berezin et al., 2011; Shamsipur et al., 2019).

**Figure 5:**
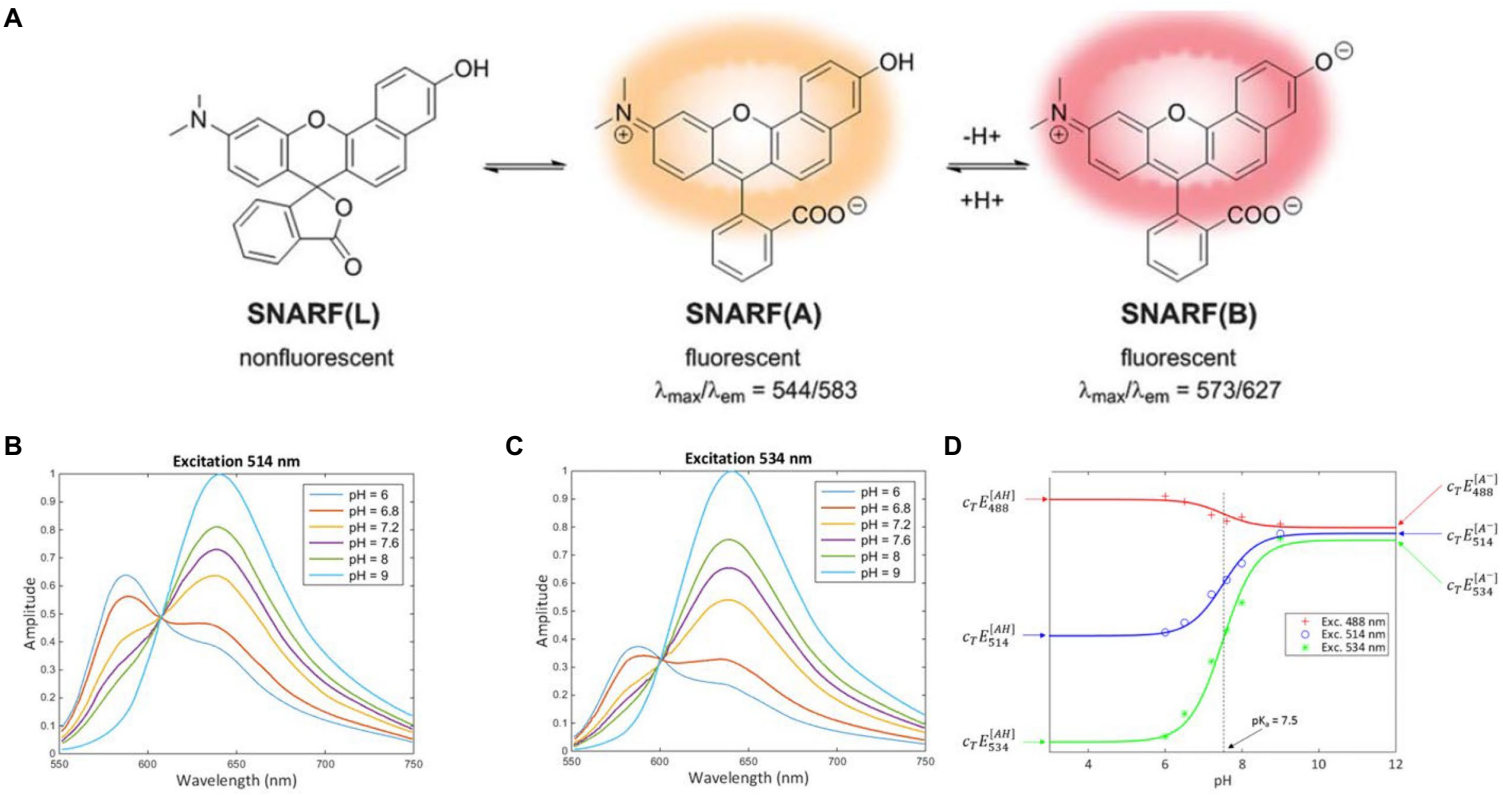
Fluorescent pH indicator dye sensing mechanism. **(A)** pH-dependent molecular equilibria structures of pH indicator dye SNARF derivative (SNARF-OH; Nakata et al., 2014). **(B)** Fluorescence emission spectra of C-SNARF-1 (5-(and-6)-Carboxy SNARF®-1) at 514 nm (Kateklum et al., 2018). **(C)** Emission spectra of C-SNARF-1 at 534 nm (Kateklum et al., 2018). **(D)** Mathematical modeling of C-SNARF-1 emission wavelength as a function of pH (Kateklum et al., 2018). These figures were reproduced with permission from Nakata et al. (2014) and Kateklum et al. (2018).

The performance of fluorescence probes based on intensity measurements at a single excitation and emission wavelength, however, is influenced by fluctuations in excitation source intensities, varied emission collection efficiencies, dye concentration, photobleaching of dye, and optical path length, which makes quantification challenging (Niu et al., 2005; Han and Burgess, 2010). Ratiometric fluorescent indicators are able to overcome these limitations since they allow for pH measurements independent of dye molecule concentration (Han and Burgess, 2010; Fulaz et al., 2019). This method requires a fluorescent dye that is differentially sensitive to pH for two excitation or emission wavelengths (Han and Burgess, 2010). The ratio between the signals can be calibrated, and thus the pH-dependent shift of the dye can be used to determine pH independent of dye concentration and optical collection efficiency (assuming the background signal is small or known). For example, seminaphthorhodafluor (SNARF) and its derivatives are commonly used ratiometric pH indicators with a pK_a_ in the neutral region (Han and Burgess, 2010; Nakata et al., 2014). Figure 5A shows the chemical equilibria between the acidic (protonated) and basic (deprotonated) forms of a SNARF derivative (Nakata et al., 2014). The SNARF derivative showed maximum absorption at 544 nm (in pH 5) and 573 nm (in pH 9), and maximum emission intensities for the dye were at 583 nm in pH 5 and 627 nm in pH 9 (Nakata et al., 2014). Figures 5B,C show the fluorescence emission spectra of a commercially available SNARF derivative as a function of pH for two different excitation wavelengths (Kateklum et al., 2018). Since the equilibrium ratio of protonated and deprotonated species depends upon the local pH according to the Henderson–Hasselbalch equation, the expected pH-dependent fluorescence for different fluorescence emission wavelengths can be modeled, and the results correspond well with the experimental data (Figure 5D). Generally, the dyes function well within 1 pH unit above or below the dye’s pK_a_ but are not sensitive for larger pH ranges. The use of fluorescent dyes enables imaging of a large region of the biofilm simultaneously, without damaging the structure of the biofilm, and does not typically drift compared to microelectrodes. Ratiometric or lifetime fluorescent measurements solve the issue of intensity depending on both pH and dye concentration because increased dye concentration increases both peaks equally.

**Figure 6:**
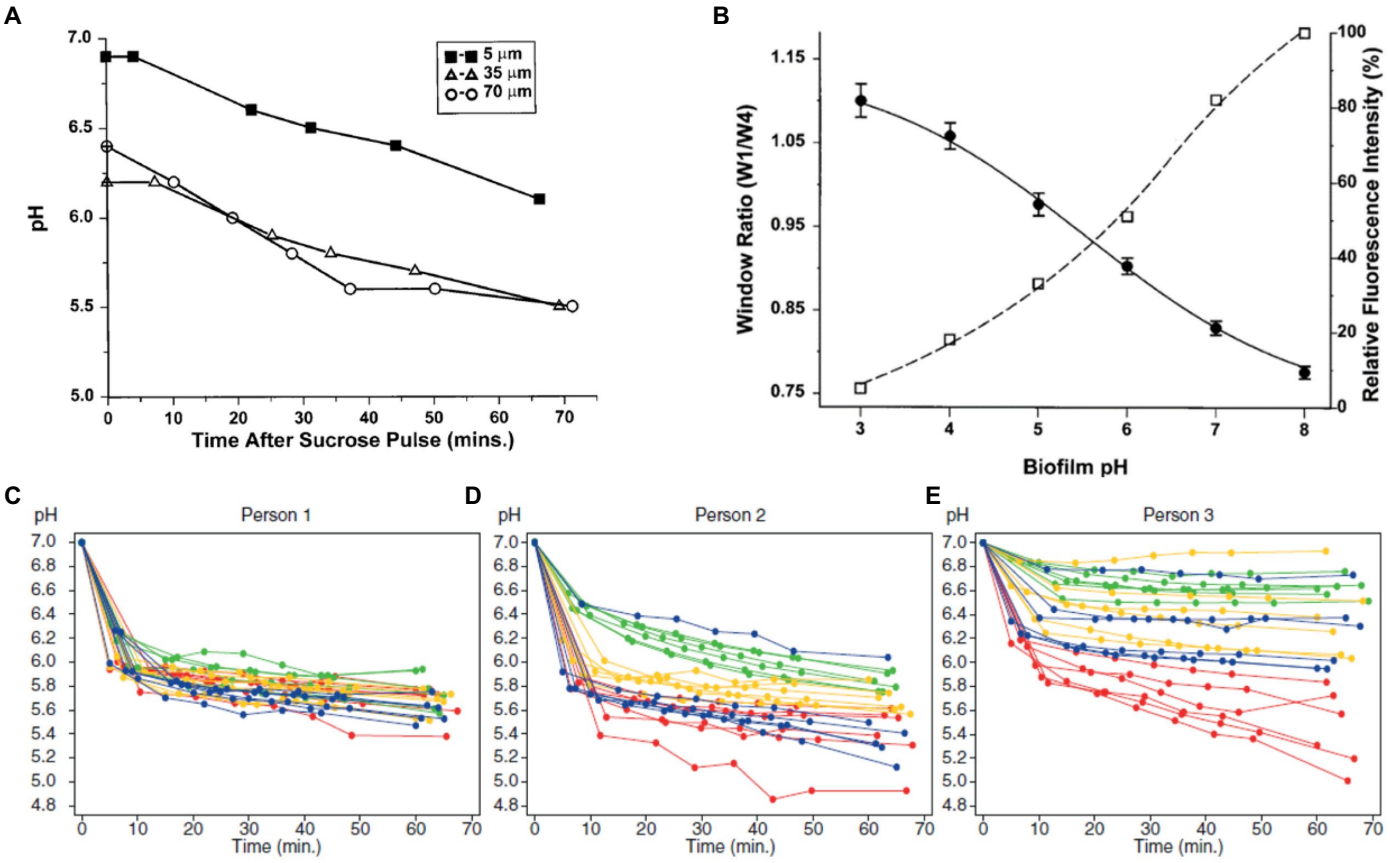
Time-dependent pH in oral biofilm after sucrose addition. **(A)** In a mixed culture of 10 oral bacteria grown in a chemostat system, the mean biofilm pH in a 100-μm biofilm decreased following a sucrose pulse at depths of 5, 35, and 90 μm from biofilm-coverslip assembly (Vroom et al., 1999). **(B)** Graph of window ratio (W_1_/W_4_) vs. biofilm pH (solid line); graph of relative fluorescence intensity of carboxyfluorescein as a function of pH (dashed line; Vroom et al., 1999). **(C–E)** Extracellular pH curves of three individuals with different pH drop patterns in the sucrose-free group from 1-h experiment. Each curve shows the average pH of a field of view; different specimens are represented by the different colors (depth of biofilm: 10–35 μm; Dige et al., 2016). These figures were reproduced with permission from Vroom et al. (1999) and Dige et al. (2016).

In a study by Vroom et al., the pH gradient within a mixed culture biofilm of oral bacteria was determined by fluorescence lifetime imaging of carboxyfluorescein indicator dye (Vroom et al., 1999). The images showed that the average pH in a polymicrobial biofilm at a depth of 5 μm is around pH 7.0 ± 0.3 and is around pH 6.0 ± 0.4 on average at a depth of 70 μm (Figure 6A). After overlaying the biofilm with sucrose, the pH in the biofilm dropped to 5.5 in 1 h due to fermentation of the sugar (Vroom et al., 1999). The graph in Figure 6A shows the decrease in pH as a result of treatment with sucrose, even at depths of 70 μm. Oral biofilm pH can also be measured by fluorescence lifetime imaging using two-photon microscopy (Dige et al., 2016). Two-photon microscopy involves excitation of a fluorophore through simultaneous absorption of lower energy two photons and has the advantage of providing high 3-D spatial resolution and low background (Benninger and Piston, 2013). Dige and co-workers used two-photon microscopy to excite the fluorescent indicator dye carboxyfluorescein in an oral biofilm and measured its emission lifetime to determine pH (Dige et al., 2016). This method is based on time-gated detection of the fluorescence for a monoexponential decay, as shown in Figure 6B. Fluorescence is detected in two-time windows after excitation of fluorochromes with the laser pulse. Fluorescence decay time can be measured by calculating the ratio of intensities, I_A_/I_B_. The technique measures fluorescence decay instead of fluorescence emission intensity, enabling concentration-dependent measurement in real-time. They found that the pH in the biofilm at a depth of 10–35 μm decreased over time after adding a glucose pulse, with most of the response occurring within 10 min but with significant variation between regions and patients.

**Figure 7:**
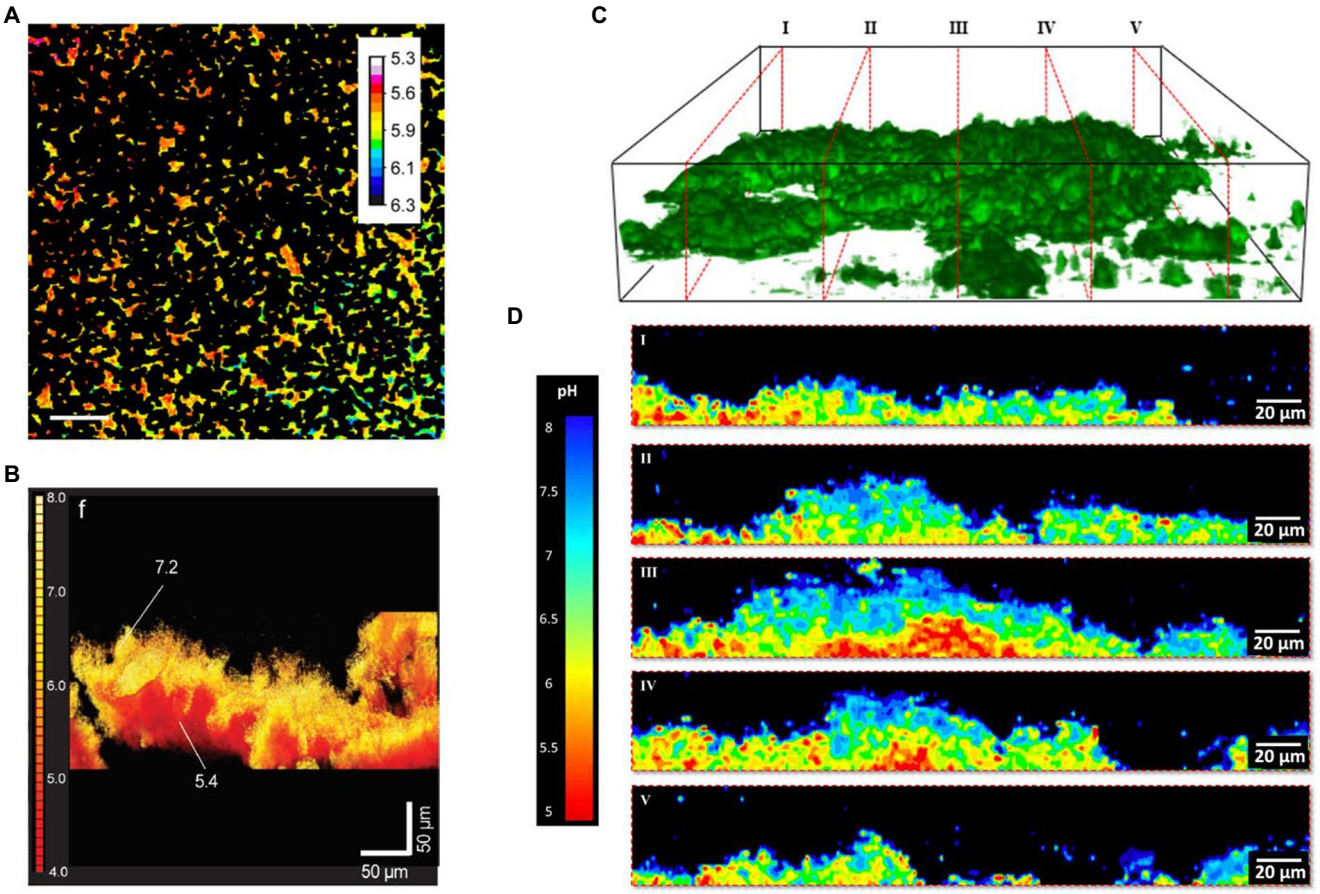
pH variation in biofilms measured *via* fluorescence. **(A)** Extracellular pH landscape in the model biofilm exposed to sterile saliva with 0.4% (w/v) glucose and C-SNARF-4 (pH-sensitive ratiometric probe). The scale bar represents 20 μm (Schlafer et al., 2011). **(B)** Functional 3-D tomographic imaging of side volumetric projections of the reconstructed biofilm pH map of *E. coli* (Hidalgo et al., 2009). **(C)** Representative 3D CLSM reconstruction of axial pH distribution within intact microcolony of *P. fluorescens* biofilms, showing orthogonal slices I-V (Fulaz et al., 2019). **(D)** Corresponding ratiometric orthogonal slices from **(C)** showing spatially distinct pH gradients in the y, z direction within microcolonies of *P. fluorescens* biofilms. Scale bars represent 20 μm (Fulaz et al., 2019). These figures were reproduced with permission from Schlafer et al. (2011), Hidalgo et al. (2009), and Fulaz et al. (2019).

Extracellular pH dropped in all biofilms from the sucrose-free group in Dige’s study after the addition of saliva containing 0.4% glucose (Dige et al., 2016). They also showed that the pH changes are time-dependent. The pH drops differ between sites from different individuals, between sites from different specimens, and within single biofilms grown on one glass slab. Figures 6C–E shows extracellular pH curves from three individuals with different pH drop patterns over the 60-min analysis experiment (Dige et al., 2016). Similarly, Schlafer and coworkers designed a five-species laboratory biofilm model and imaged it using pH-sensitive ratiometric probe Seminaphtorhodafluor-4F 5-(and-6) carboxylic acid (C-SNARF-4) to analyze the extracellular pH landscape at the interface between bacterial biofilm and underlying substrate (Figure 7A; Schlafer et al., 2011). They were able to visualize horizontal pH gradients in the young dental model biofilms. After the addition of 0.4% glucose, the pH dropped differently in various regions, from pH 7 to pH 5.5–5.9, up to a depth of 70 μm in the biofilm (Schlafer et al., 2011). Although fluorescence imaging of the biofilm can provide good quality images of pH spectra and a 3D view of the pH imaged in the biofilm, there can be issues with possible interference of the dye with cell proteins.

These results generally show that the pH within biofilms decreases when stimulated with glucose due to the production of various organic acids by the fermentation of glucose by biofilm microorganisms. In addition, the studies demonstrate the pH heterogeneity in biofilm models due to different rates of acid production and oxygen depletion within the biofilm microenvironment.

However, several fluorescent dyes have some limitations. The dyes may interact with the biofilm by binding to proteins in cells and the extracellular matrix, which can affect their effective pK_a_ and spectrum. Ideally, these also should be calibrated *in situ*, especially for absolute values. These dyes may also undergo quenching, and photobleaching, resulting in cellular toxicity and limited brightness (Hidalgo et al., 2009). In addition, confocal microscopy and two-photon scanning microscopy, which are needed to obtain spatial information on pH distribution within a biofilm, are time-consuming and require specialized equipment and personnel (Benninger and Piston, 2013; Merkl et al., 2021).

### Fluorescent nanoparticles

Fluorescent nanoparticle-based systems can be used to probe biofilm pH depending on the pK_a_ of the dye. To insulate the dye from large proteins that can affect its pK_a_ and performance, the dye can be encapsulated in nanoparticles. These consist of pH-sensitive dyes bound to nano-sized polymer matrices resulting in fast, bright responses that can be measured using real-time fluorescence microscopy (Hollmann et al., 2021). Usually, a reference dye with an emission intensity insensitive to pH is also incorporated into the polymer matrix to enhance signal stability (Niu et al., 2005). Several different pH dyes can be combined into a single nanoparticle, enabling measurement in a range of pH, and targeting is possible by controlling the surface functionalization of the nanoparticles (Fulaz et al., 2019). Similarly, other analytes can be detected fairly easily as well since they allow the attachment of multiple components. This method provides a robust system for sensing *in situ* pH gradient in a biofilm without disrupting the biofilm structure. The polymer matrix insulates the dye from the environment, which reduces interference from adsorption to proteins such as BSA, reduces concerns on toxicity and solubility, and improves their photostability (Shamsipur et al., 2019). A major drawback of this method is that pH-sensitive nanoparticles are not typically available commercially. Further, nanoparticles are larger than dyes, which affects penetration into the biofilm and can be limited by low diffusion rates (Hidalgo et al., 2009). The dye can also leach over the long term if not covalently bound. Another drawback would be that based on surface chemistry, size, and biofilm, their intracellular and extracellular distribution will be different. Due to encapsulation of the dye, the calibration curve dye changes from its free form due to the changes in its local environment. In addition, the curve may also be stretched due to the presence of multiple environments within the nanoparticle resulting in response over a larger pH range but with less sensitivity.

For example, Hidalgo and coworkers used ratiometric fluorescent silica nanoparticle sensors as tools for high-resolution 3D and time-domain functional fluorescence imaging of pH gradients in microbial biofilms (Hidalgo et al., 2009). Their study showed pH values ranged from the acidic core (pH 5) to the neutral outside surface of biofilm (pH > 7), confirming the heterogeneity of the pH profiles within the water waste biofilms (Figure 7B). Adding glucose resulted in a more acidic pH due to glucose metabolism causing the release of tricarboxylic acid cycle acids and CO_2_ (_Hidalgo et al.,__2009_). Changes in pH of dental biofilm in response to 0.4% glucose are studied with ratiometric pH-sensitive dye C-SNARF-4 with digital image analysis.

In another study by Fulaz et al., pH-responsive nanoparticle sensors were used to image through the biofilm (Fulaz et al., 2019). The sensors made of mesoporous silica nanoparticles conjugated to fluorescein (pH-sensitive dye) and rhodamine-B (pH insensitive dye) were able to penetrate through the *Pseudomonas fluorescens* biofilm and enable ratiometric analysis of pH gradients. As shown in Figures 7C,D, confocal laser scanning microscopy (CLSM) images show distinct heterogeneous regions in the biofilm, with more acidic regions within the biofilm compared to the outer regions.

Table 1 summarizes the pH measurement methods for determining the biofilm pH gradient and the advantages and disadvantages of those methods. Table 2 lists the studies on the pH gradient in biofilms, their results, and the effect of carbon sources.

**Table 2:** Studies on pH gradient in biofilm and the effect of carbon source.

Study method		Biofilm type	pH Range	Findings	Effect of carbon source	References
pH microelectrodes		Monospecies *Citrobacter* sp. biofilms (can be found in food and water)	From pH around 7 to the lowest pH of 5.12	The pH in phosphate-limited biofilm fell within the biofilm (pH shift of 0.4 and 1.2 pH units in 0–400 μm from the substratum), whereas that of carbon-limited biofilm remained constant.	Biofilm on phosphate-limiting medium became more acidic.	Allan et al. (1999)
Fluorescent dyes	C-SNARF-4 confocal microscopy	Dental biofilm multi-species	7 to 5.5–5.9	Heterogeneous pH in the basal layer of young dental biofilm model (7–100 μm), no obvious correlation between biofilm thickness and pH drop in the bottom layer.	Adding glucose caused pH drop.	Schlafer et al. (2011)
			7 to 4.5	Highly heterogeneous pH landscapes due to supply of glucose and buffering ions, bacterial acid production, bacterial acid consumption, and diffusion of acids within and out of biofilm.	The pH dropped in deeper layers of five-species laboratory biofilms (up to 70 μm) upon exposure to 0.4% glucose.	Dige et al. (2016)
	*TPE and CLSM with fluorescence lifetime imaging	Mixed culture oral biofilm	Within 10 min of exposure to glucose, pH fell from 7.0 ± 0.3 to 6.0 ± 0.4	Sharp gradients of pH within microbial biofilms.	Adding sucrose resulted in pH drop, especially in layers at depths of 35 and 70 μm.	Vroom et al. (1999)
Fluorescent nanoparticles	Silica nanoparticle sensors confocal microscopy	Mixed-culture wastewater biofilms	5 to >7	Heterogeneity of the pH profiles within these biofilms. The pH is more heterogeneous throughout the mixed culture than the axenic one at 77 μm from the substratum.	By adding glucose, pH becomes more acidic.	Hidalgo et al. (2009)
	pH-responsive nanoparticles confocal microscopy	*P. aeruginosa* (opportunistic pathogen) and *Streptococcus* mutants (oral pathogen)	3.5 to 7.5	pH gradient across the biofilm thickness and over time and an acidic core of microcolonies.	pH dropped after the addition of carbon sources (glucose and sucrose).	Hollmann et al. (2021)
		*P. fluorescens* (soil and water)	5 to 8	pH gradient at different depths of biofilm (0–40 μm).	Biofilm produced more acidic by-products under oxygen-limiting conditions found in the deepest regions of the biofilm.	Fulaz et al. (2019)

* Two-photon excitation microscopy (TPE) and conventional confocal laser scanning microscopy (CLSM).

## Bacterial infection diagnosis

Clinical diagnosis of bacterial infection on implant surfaces is mainly based on signs and symptoms of infection, such as inflammation or pain (Campoccia et al., 2006). The criteria of these diagnosis methods are not standard and can result in false positive or false negative detection of infection (Campoccia et al., 2006; VanEpps and Younger, 2016). The existing diagnosis methods are divided into culture-based and non-culture-based methods. Tissue samples and synovial fluid are cultured to confirm the presence of pathogens. Polymerase chain reaction (PCR) is a non-culture-based method and a molecular pathogen-based technique being used to identify the pathogen. Tracking inflammatory cytokine biomarkers like procalcitonin (PCT), interleukins (IL-6 and IL-1ß), and alpha-defensin are also non-culture-based methods (VanEpps and Younger, 2016). These methods are time-consuming and require expensive equipment and specialized personnel. In addition, bacteria colonizing the implant surface forming biofilms will be resistant to antibiotics and the host’s immune system, and the diagnostic tests are performed only upon the development of infection symptoms (Stewart and Costerton, 2001; VanEpps and Younger, 2016). None of the currently available diagnosis methods provide enough sensitivity, specificity, and simplicity for early and effective noninvasive detection of microenvironment changes around implant surfaces (Vasoo, 2018). If these infections are not detected early, surgical debridement and device removal will be necessary to treat infections. Therefore, it is necessary to develop fast, cost-effective, and accurate methods to effectively detect and treat implant infections as early as possible.

**Figure 8:**
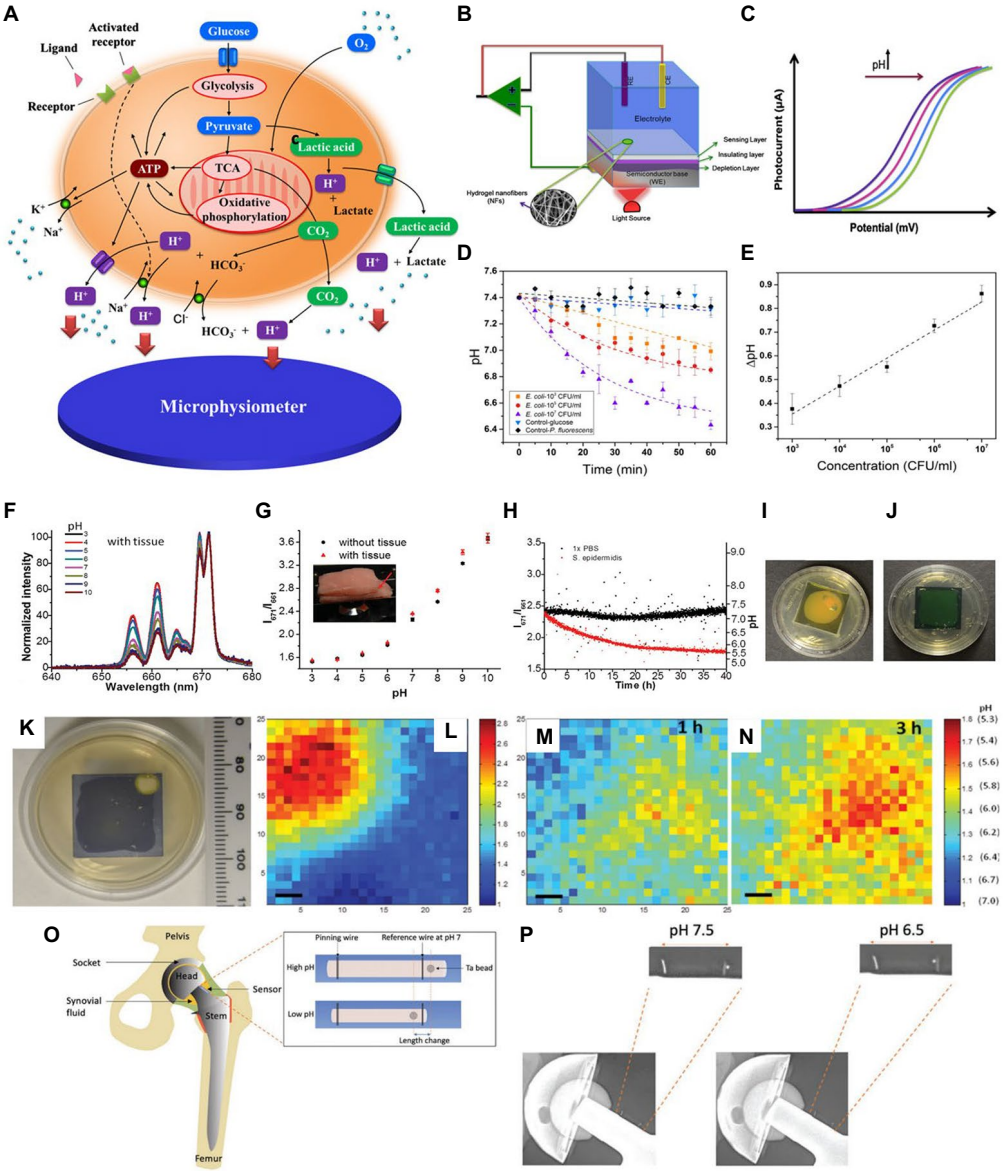
Different approaches to measuring local biofilm pH. **(A)** Principle of cellular metabolism pathways resulting in extracellular acidification, which can be measured using pH as a microphysiometer (Hu et al., 2013). **(B)** Schematic of a light addressable potentiometric sensor (LAPS) with hydrogel nanofibers (NFs) as part of a three-electrode electrochemical sensing and imaging system; working electrode (WE), refere1nce electrode (RE), counter electrode (RE; Shaibani et al., 2016). **(C)** A typical response of LAPS to changes in pH for a p-type semiconductor (Shaibani et al., 2016). **(D)** Variation of pH over time at different *E. coli* concentrations measured with LAPS (Shaibani et al., 2016). **(E)** Changes in pH versus *E. coli* concentrations measured with LAPS (Shaibani et al., 2016). **(F,G)** pH measurement using an implantable upconversion particle sensor film (Wang et al., 2013). **(F)** Luminescence spectra of pH sensor film after passing through porcine tissue in standard pH buffers, normalized to peak at 671 nm (Wang et al., 2013). **(G)** pH response of pH sensor film (black dots – no porcine tissue; red triangles – passing through porcine tissue). Inset shows a photograph of the pH sensor film between porcine tissue, with the red arrow showing the pH sensor (Wang et al., 2013). **(H)** Real-time pH monitoring with pH sensor film through tissue (red dots indicate pH variation due to *S. epidermis* growth; black dots indicate pH variation due to the control, which was phosphate buffer saline; Wang et al., 2013). **(I)** Image of pH sensor film inoculated with *S. epidermidis* after acquisition (Wang et al., 2013). **(J)** Image of pH sensor film control sample after acquisition (Wang et al., 2013). **(K)** Image of the sample *S. epid.* 12,228 at pH sensor film and TSA plate interface at 37°C (Wang et al., 2015). **(L–N)** pH mapping using XELCI. Peak intensity at 620 nm over intensity at 700 nm is represented by the color bar. Step size is 300 μm; the scale bars represent 1 mm **(L)** pH ratio map of the reference region through XELCI (Wang et al., 2015). **(M)** pH ratio map of sample region through XELCI (after 1 h growth; Wang et al., 2015). **(N)** pH ratio map of sample region through XELCI (after 3 h growth; Wang et al., 2015). **(O)** Schematic of the prosthetic hip implant with attached pH sensor. Inset shows the mechanism of pH sensing (Wijayaratna et al., 2021). **(P)** X-ray images of pH sensor at pH 6.5 and 7.5 in bovine synovial fluid (Wijayaratna et al., 2021).

As discussed above, biofilms generate acidic regions which both correlate with antibiotic-resistant regions and influence local biochemistry. Measuring and imaging local pH near the implant surface, where the persistent and antibiotic-resistant bacteria reside, would be useful for both detecting/monitoring infections and understanding the environment in order to develop treatments for these resistant regions. It is necessary to develop ways to measure the local pH since the pH can change over 20 μm distances (Figure 7). Sensing systems based on the light-addressable potentiometric sensor (LAPS) provide a simple platform to measure pH locally (Hafeman et al., 1988; Owicki et al., 1994; Adami et al., 1995). Figure 8A shows the principal metabolic pathways that lead to extracellular acidification (Hu et al., 2013). Anaerobic respiration of bacteria results in the production and accumulation of acidic byproducts. This extracellular acidification can be measured using a LAPS-based instrument called a microphysiometer. The LAPS can be considered as a working electrode in a three-electrode electrochemical setup consisting of a working electrode, reference electrode, and counter electrode (Shaibani et al., 2016; Figure 8B). LAPS consists of a photoconductive substrate, an insulating layer, and a sensing layer in contact with an electrolyte (usually silicon nitride). When the sensor chip is illuminated, electron–hole pairs are generated in the photoconductor layer, resulting in a localized “short circuit,” where current and voltage can be applied and measured to determine pH. The typical LAPS response varies with pH, as shown in Figure 8C, for a p-type semiconductor photoconducting layer, with the photocurrent shifting to higher potential values with an increase in pH (Shaibani et al., 2016). In the study by Shaibani et al., the sensing layer is composed of polyacrylic acid/ polyvinyl alcohol nanofibers. They were able to detect changes in pH in the media upon sugar fermentation by *E. coli* (Figure 8D). A linear response was observed from the graph showing the change in pH at different concentrations of *E. coli* after 1 h, with a detection limit of 20 CFU/ml (Figure 8E). This method provides an inexpensive, rapid method for measuring pH. However, LAPS is currently limited to *in vitro* measurements, as it requires addressable light source and complex electronics to operate, and those are currently impractical to miniaturize and apply to medical implant surfaces. In order to measure pH near implant surfaces, it is necessary to develop passive devices and coatings with wireless readout.

**Figure 9:**
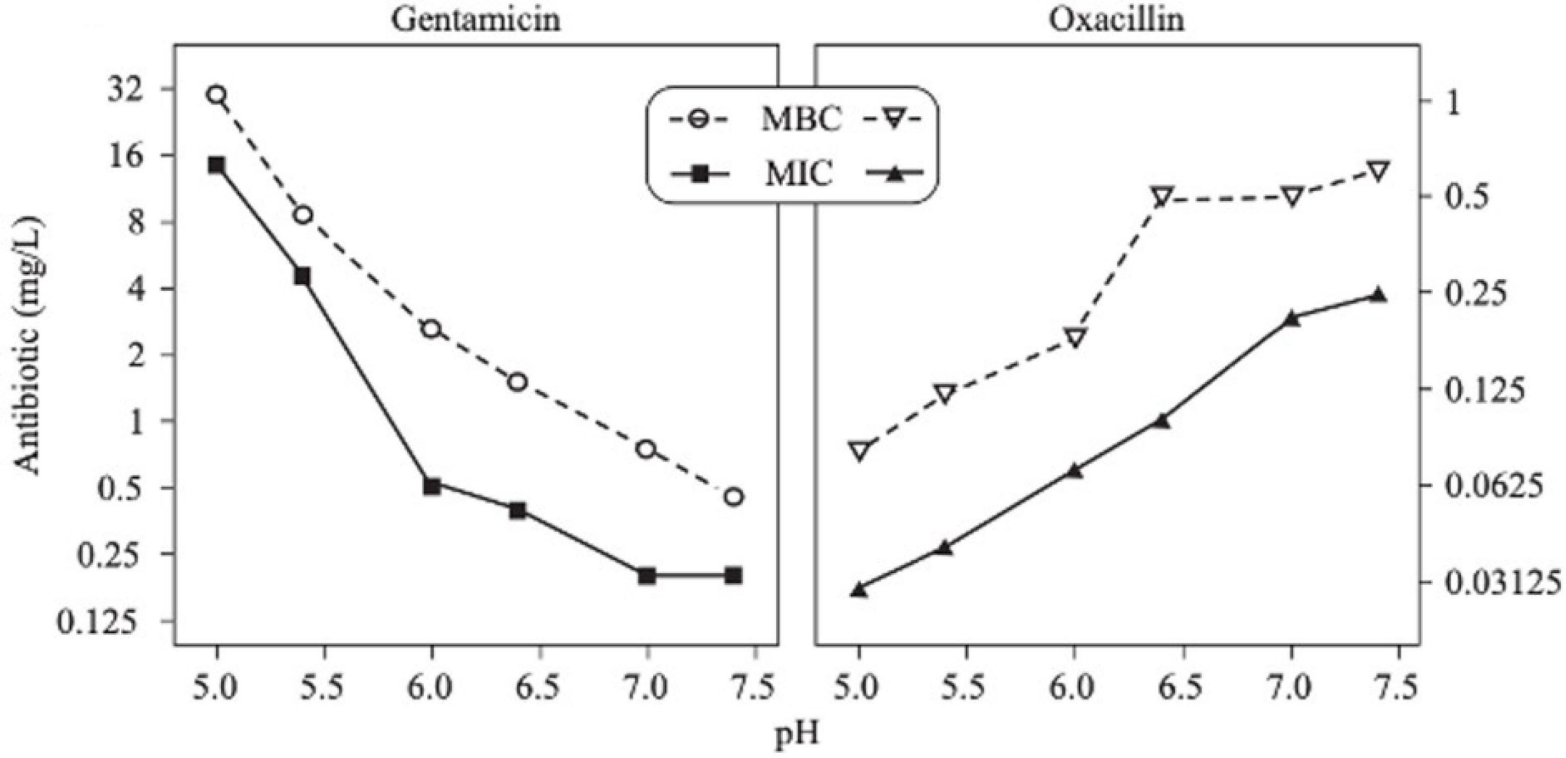
MICs and MBCs of two antibiotics (gentamicin and oxacillin) as a function of pH on *S.* aureus (Baudoux et al., 2006).

These figures were reproduced with permission from Hu et al. (2013), Shaibani et al. (2016), Wang et al. (2013), Wang et al. (2015), Florence and Attwood (2007), and Wijayaratna et al. (2021).

Biomedical sensors responsive to pH can be used for detecting and monitoring biofilm formation and bacterial infection on orthopedic implants. A sensor based on a film consisting of upconverting particles (UCPs), which act as a light source, and pH indicator dye (bromocresol green) can be used to measure local pH (Wang et al., 2013). Irradiation of the film with 980 nm light causes the UCPs to emit red light, and the indicator dye would absorb more at 661 nm than at 671 nm, and the resulting spectral ratio would indicate pH. The luminescence spectra of the pH sensor at different pHs under porcine tissue are shown in Figure 8F, and it was observed that the presence of tissue did not have a significant effect on the calibration graph of the sensor (Figure 8G; Wang et al., 2013). The pH sensor film was used to study the variation in pH due to bacterial growth of *Staphylococcus epidermis,* which showed that the pH decreased gradually after the inoculation of bacteria (Figure 8H). The image of the TSA plate after acquisition for the bacteria-inoculated sample showed colonies of bacteria growing on the edges of the pH sensor with a change in color of the film from green to yellow indicating acidosis (Figure 8I). In contrast, as expected, the control TSA plate remained green and did not have any bacteria growing on it (Figure 8J).

X-ray excited luminescent chemical imaging (XELCI) is a novel medical imaging technique used to non-invasively detect and monitor bacterial biofilm on implant surfaces (Wang et al., 2015; Uzair et al., 2020). In the XELCI technique, a combination of X-ray excitation to provide high-resolution images and optical detection of infection is used. A layer of X-ray scintillators that generate visible near-infrared light when irradiated with an X-ray beam is used to coat the implant. The light first passes through a pH indicator dye-loaded film placed over the scintillator film to modulate the luminescence spectrum according to pH. The light that passes through tissue is then collected, and the spectral ratio is measured to determine the pH. A focused X-ray beam irradiates a point in the scintillator film, and a pH image is formed point-by-point by scanning the beam across the sample (Uzair et al., 2020). A pH sensor film was developed by Wang et al. and can be coated on implant surfaces to noninvasively map pH *ex vivo* in porcine tissue using XELCI (Wang et al., 2015). The pH map showed a decrease in pH due to bacterial growth on the interface between the pH sensor film and the tryptic soy agar (TSA) plate (Figures 8K–N). A similar pH sensor attached to implants was developed to noninvasively detect local implant pH. Both these sensors enabled noninvasive mapping of local pH, with high spectral resolution (Wang et al., 2015; Uzair et al., 2019, 2020). A hydrogel sensor that can be attached to a prosthetic hip implant is able to measure synovial fluid pH using plain radiography (Wijayaratna et al., 2021). The sensor consists of a pH-responsive hydrogel with a radiodense tantalum bead and a metal wire at the two ends. Once the sensor is attached to the prosthetic implant, the change in length of the hydrogel in response to the pH of synovial fluid can be monitored *via* plain radiography (Figure 8O). X-ray images of the sensor attached to a hip implant clearly show the radiopaque markers, and the pH response can be determined from the radiographs by measuring the movement of the tantalum bead with respect to the radio-opaque metal wire (Figure 8P). This would enable simple, noninvasive pH measurements *in vivo* at the site of infection.

## Effect of pH

### Effect of pH on biofilm

The majority of biofilm infections on implant surfaces are caused by staphylococci, of which *S. aureus* and *S. epidermidis* are the most common causative agents (Campoccia et al., 2006). A study by Stewart et al. showed variation in the distribution of *S. aureus* and *S. epidermidis* at different pHs (Stewart et al., 2017). In a multiple species biofilm, *S. epidermidis* was seen to grow more at low pH conditions such as pH 5 and 6 compared to neutral pH. At higher pH values (pH 7, 8, and 9), *S. aureus* was the dominant species in the biofilm. *S. epidermidis* did not incorporate well into the biofilm at high pH and was seen to remain as individual cells or clusters of cells. The dominant behavior of *S. epidermidis* at low pH may be due to its ability to survive in low pH conditions since they typically reside in the skin with low pH conditions ranging from pH 4.0 to 7.0 (Lambers et al., 2006; Stewart et al., 2017). Another study by Stewart et al. showed that the biofilms of both *S. aureus* and *S. epidermis* soften at high pH (>7), which can be used as a potential method to disrupt staphylococcal biofilms (Stewart et al., 2015). Hostacka et al. studied the effect of pH on other pathogens related to infections, including *P. aeruginosa*, *Klebsiella* spp., and *V. cholerae* (Hoštacká et al., 2010). These microorganisms demonstrated an increase in biofilm production at higher pHs (pH 7.5 and 8.5) compared to pH 5.5. Therefore, the effect of pH on the biofilm depends mainly on the type of species in the biofilm.

Low pH can affect the extracellular matrix material (polysaccharides, proteins, and DNA) and cellular interactions with it. For example, Foulston et al. reported that extracellular proteins produced by *S. aureus* (or exogenously added) attached to the bacteria in a pH-dependent manner, and that biofilms were not formed at pH 7.5 (Foulston et al., 2014). Similarly, Antikainen et al. found a pH-dependent association of proteins such as glyceraldehyde-3-phosphate dehydrogenase (GAPDH) and enolase attachment to the surface of *Lactobacillus crispatus* (Antikainen et al., 2007). Foulston hypothesized that the low pH may have caused the proteins to become positively charged and better associated with negatively charged molecules on the cells and extracellular DNA (Foulston et al., 2014). They also suggested that the need for low pH may contribute to the increase in biofilm production at high glucose concentrations. While more studies are needed, these results show that pH can influence biofilm growth and behavior in multiple ways.

### Effect of pH on host

The effect of local acidosis at the site of infection on the immune system directly influences the functions of immune cells. Studies on polymorphonuclear leukocytes show impaired chemotaxis, respiratory activity, and bactericidal capacity at lower pH (Zigmond, 1978; Lardner, 2001; Kellum et al., 2004; Olmsted et al., 2005). Clinical acidosis is usually accompanied by immunodeficiency characterized by severe neutropenia, lymphopenia, and infection (Brandt, 1984; Wong et al., 1992). Some of the other effects on the immune system include activation of oxygen burst in neutrophils, production of reactive oxygen species, neutrophil phagocytosis, and neutrophil apoptosis (Gabig et al., 1979; Nakagawara et al., 1981; Leblebicioglu and Walters, 1999; Trevani et al., 1999). A positive effect of low pH on immune function has been shown with acid-induced activation of complement proteins and alternative complement pathways (Hammer et al., 1983; Miyazawa and Inoue, 1990; Emeis et al., 1998). Increased antibody-binding to leukocytes at lowered pHs due to pH-dependent conformational change has been demonstrated in a study by Miyazawa and Inoue (Miyazawa and Inoue, 1990).

It has been found that the F-ATPases from oral lactic-acid bacteria can play a role as ATP synthases and lead to a decrease in pH. These bacteria use glycolysis as their ATP source for survival, and it protects these bacteria from being killed in the lethal acidic pH environment (Bender et al., 1986; Sturr and Marquis, 1992; Sheng and Marquis, 2006). In addition, acid tolerance of bacteria depends on the rate at which ATP can be supplied from the catabolism of various substrates (Belli and Marquis, 1994).

Studies show that oxygen levels may affect resistance to infection of some bacteria. Since areas of low oxygen levels in biofilms are closely linked to low pH, it is expected that the same effects may be seen in low pH regions as well. In a study by Jönsson et al., high oxygen levels decreased the extent of test infections by *S. aureus*, whereas a reduced oxygen supply had the opposite effect, especially when tissue pO_2_ was below 40 mmHg (Jönsson et al., 1988). *In vitro* studies demonstrated that low oxygen levels (in the range of 0–40 mmHg) reduced the rate at which leukocytes generated oxygen radicals, which help kill bacteria (Hunt and Pai, 1972; Erra Díaz et al., 2018). Simultaneously, carbon dioxide levels may also affect the activity and function of neutrophils. Neutrophils at the low pCO_2_ sites had a higher inflammatory response than intracellular acidification caused by high pCO_2_ (Coakley et al., 2002). Therefore, acidification of the environment surrounding the immune cells affects immune responses and healing of infections since chronic inflammation delays the healing progress (Lardner, 2001).

### Effect of pH on antibiotic activity

The activity of some antibiotics is highly pH-dependent and depends on their structure and mode of action. An antibiotic’s susceptibility and resistance are measured by the minimum inhibitory concentration (MIC) and minimum bactericidal concentrations (MBCs; Andrews, 2001). MIC is defined as the lowest concentration of an antimicrobial that will inhibit the visible growth of a microorganism after overnight incubation. MBC is the lowest concentration of an antimicrobial that will prevent the growth of an organism after subculturing onto antibiotic-free media. Therefore, the acidic pH of biofilms will affect the antibiotic activity and, in turn, affect the therapeutic response to a particular drug (Cunha, 2016). For three classes of antibiotics, lincosamides, macrolides, and aminoglycosides, lowering the pH has been shown to increase their MICs (Table 3). Hinnu et al. (2022), demonstrated that the MIC of Azithromycin, a broad-spectrum macrolide used for treating invasive salmonellosis, can be greatly influenced by pH shifts. Under different buffered conditions, the MIC values could vary almost 1,000 folds. In a study by Baudoux et al. (2006), the MIC and MBC of gentamicin (an aminoglycoside class antibiotic) increased with a decrease in pH, where the MIC was approximately 70 times higher at pH 5 than at pH 7.4 (Figure 8). Acidic pH was shown to drastically reduce the activity of gentamicin (Ward and Steigbigel, 1978). This is considered one of the main reasons for treatment failures of aminoglycosides in infections where the pH is acidic (Simmen et al., 1993) and for the poor efficacy against intracellular forms of *S. aureus* (Maurin and Raoult, 1994; Barcia-Macay et al., 2006). Conversely, acidic pH has been shown to decrease the MIC of antibiotic classes β-lactams and tetracyclines (Table 3; Lemaire et al., 2005; Yang et al., 2014). In the same study by Baudoux et al., lowering the pH increased the activity of oxacillin (a β-lactam class antibiotic), with a decrease in both MIC and MBC (Figure 8; Baudoux et al., 2006). Other studies have shown that at values lower than physiological pH, vancomycin inhibits the growth of *S. aureus* more effectively (Table 3; Barcia-Macay et al., 2006). A possible explanation may be due to the hydrolysis of β-lactams at ambient pH conditions compared to acidic pH (Mitchell et al., 2014).

**Table 3a:** Effects of pH on MIC of various classes of antibiotics.

Mode of Action	Class	Antibiotic	Optimal pH (lowest MIC)	pI	Organism	Findings	References
Peptide Synthesis Inhibition	Aminoglycoside	Gentamicin 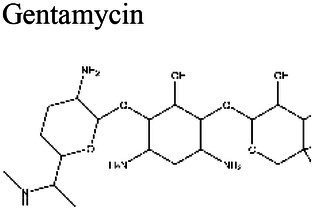	8.5 (a); 7.5 (b); 7.3 (c)	9.5 (d)	*S. aureus* (b-c)	MIC is 70x higher at pH 5 compared with pH 7.4 (b).	(a) Hancu et al. (2015), (b) Baudoux et al. (2006), (c) Barcia-Macay et al. (2006), (d) Henner and Sitrin (1984)
Peptide Elongation Inhibition	Lincosamides	Lincomycin 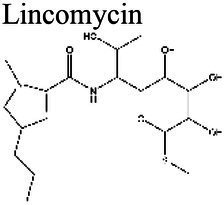	8.5 (a)	< 7.6 (b,c)	Group D Streptococcus (Enterococci) (a)	MIC is smaller at more alkaline pHs (7.4–8.5) than at 5.0 (a).	(a) Toala et al. (1970), (b) Herr et al. (1967), (c) Florence and Attwood (2007)
		Clindamycin 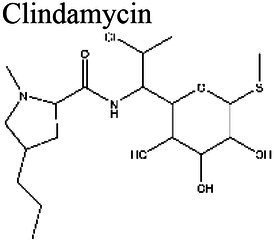	8.5 (a); > 4.5 (b)	< 7.5 (c)	Group D Streptococcus (Enterococci) (a) *Clostridium acetobutylicum* (b)	Similar MICs at pH 7.4–8.5, gets larger at acidic pH (a). At pH 4.5 the antibiotic was completely ineffective against *C. acetobutylicum* (b).	(a) Toala et al. (1970), (b) Mermelstein and Papoutsakis (1993), (c) Florence and Attwood (2007)
	Macrolides	Erythromycin 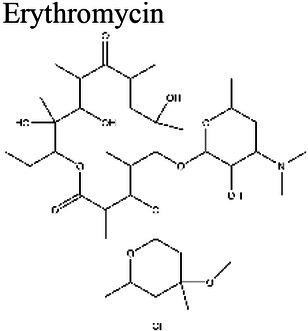	8.5 (a); 8.0 (b)	10.41 (c)	Group D Streptococcus (Enterococci) (a) *E. coli* (b)	4-fold more effective at pH 8.5 than at pH 7.4 (a); Erythromycin extremely effective at alkaline pH (8.0). Completely loses efficacy at pH ≤ 6 (b).	(a) Toala et al. (1970), (b) Lorian and Sabath (1970), (c) National Center for Biotechnology Information (2004a)
Prevention of DNA Unwinding	Quinolones	Ciprofloxacin 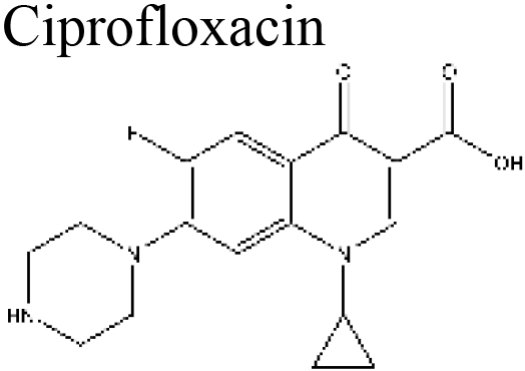	7.4–8.4 (a); 7.3 (b); 7–10 (c)	7.39 (d) 7.4 (e)	Staphylococcus (a); *S. aureus* (b) *Proteus mirabilis* (c)	Loses efficacy at acidic pH (5.4–6.4) when compared to neutral or alkaline pH (7.4–8.4) (a); 8x larger MIC at pH 5.0 compared to pH 7.4.	(a) Tato et al. (2014), (b) Barcia-Macay et al. (2006), (c) Irwin et al. (2013), (d) Comer (2007), (e) Horká et al. (2014)
		Levofloxacin 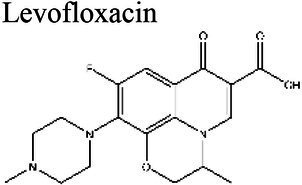	7.4–8.4 (a); 7.3 (b); 7.2 (c)	6.8 (c)	Staphylococcus (a); *S. aureus* (b) *Enterobacteriaceae, K. pneumoniae* (c)	Retains antimicrobial efficacy at neutral (7.4) or alkaline pH (8.4) but not at acidic pHs. (a); 8-fold larger MIC at pH 5.0 than at pH 7.4 (b); 4–32 times smaller MIC at pH 7.2 compared to pH 5.8 (c).	(a) Tato et al. (2014), (b) Barcia-Macay et al. (2006), (c) Dalhoff et al. (2017)
Folate Synthesis Inhibition	Sulfanolamides	Sulfanilamide 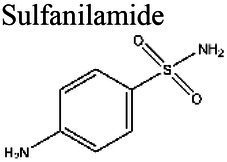		6.41			Martınez and Gomez (2001)
		Sulfadiazine 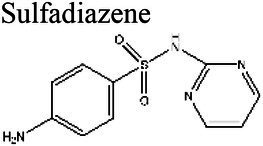	6.0 (a)	4.24 (b)	*Daphnia magna* (a)	11 times more effective at pH 6.0 than at pH 8.5 (a).	(a) Anskjær et al. (2013), (b) Martınez and Gomez (2001)
mRNA Translation Inhibition	Tetracyclines	Tetracycline 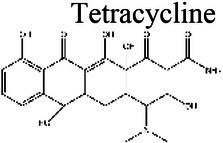	> 5.0 (a)	5.4 (b)	Group D Streptococcus (Enterococci) (a)	The MIC of tetracycline is slightly lower at pH than at pH 7.4 or 8.0 (a).	(a) Toala et al. (1970), (b) Horká et al. (2014)
		Doxycycline 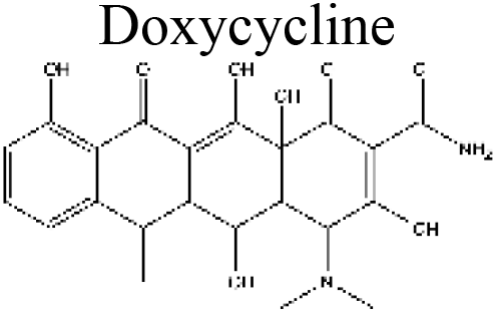	5.0 (a, b)	5.5 (c)	Group D Streptococcus (Enterococci) *E. coli, S. epidermidis* (b)	MIC at pH 5.0 was approximately 4x lower than at 7.4 (a). Increased susceptibility of *E. coli* at pH 5.0 when compared with pH 8.0 (b).	(a) Toala et al. (1970), (b) Yang et al. (2014), (c) Giovagnoli et al. (2010)

**Table 3b:** Effects of pH on MIC of various classes of antibiotics.

Mode of Action	Class	Antibiotic	Optimal pH (lowest MIC)	pI	Organism	Findings	References
Cell Wall Biosynthesis Inhibition	Penicillin	Ampicillin 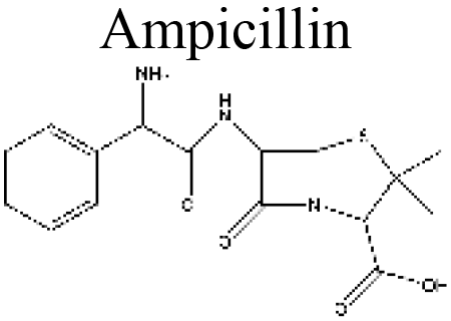	≥ 4.5 (a) 5.0 (b,c)	5.03 (d); 4.9 (e)	*Helicobacter pylori* (a); Group D Streptococcus (Enterococci) (b); *S. aureus* (c)	At lower pH (3.0), ampicillin ceased to be effective against *Helicobacter* due to the non-replicative state of the bacteria (a). At lower pHs, the efficacy of ampicillin against Gram-positive Enterococcus increased relative to alkaline pHs (b). In human serum, ampicillin had increased efficacy against *S. aureus* at lower pHs (c).	(a) Marcus et al. (2012), (b) Toala et al. (1970), (c) Barcia-Macay et al. (2006), (d) Bezerra et al. (2018), (e) Horká et al. (2014)
		Amoxicillin 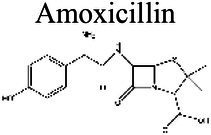	6.0 (a)	4.7 (b)	*E. coli* (a)	100% of tested ampicillin-resistant *E. coli* strains were susceptible to amoxicillin at pH 6 compared to only 28% resistance at pH 7.4 (a).	(a) Cunha (2016), (b) Feng et al. (2006)
		Oxacillin 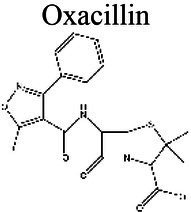	5.5 (a); 5.0 (b)	1.82 (c)	*S. aureus*	At pH 5.0, the MIC is ten times lower than at pH 7.4 (a). At pH 5.0 in blood serum, the MIC is lower than at 7.4 (b).	(a) Baudoux et al. (2006), (b) Barcia-Macay et al. (2006), (c) National Center for Biotechnology Information (2004b)
	Cephalosporins	Ceftriaxone 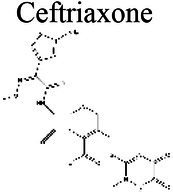		3.68			Hancu et al. (2015)
		Cephalothin 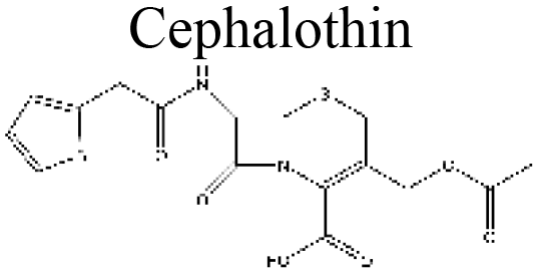	5.0 (a)	<3.8 (b)		At pH 7.4–8.0, the MIC is 5x larger than at pH 5.0 (a).	(a) Toala et al. (1970), (b) National Center for Biotechnology Information (2004c)
	Carbapenems	Meropenems 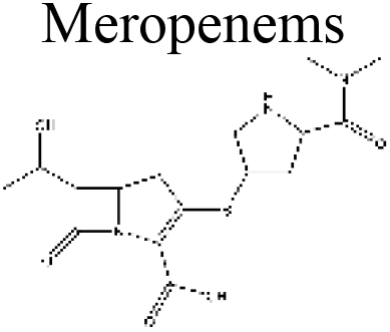	5.8–7.1 (a)	5.15 (b)	*B. fragilis* (a)	At a range of pH 5.8–7.1, the change in MIC only slightly increased at lower pHs (a).	(a) Falagas et al. (1997), (b) Blumer (1997)
	Glycopeptides	Vancomycin 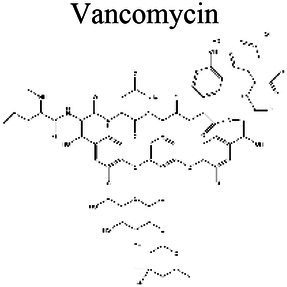	6.4 (a); 5.0–7.3 (b)	8.1 (c,d)	*S. aureus* (a-b)	Vancomycin demonstrated on average slightly smaller MICs at a low pH of 6.4 vs. 7.4–8 (a). In blood serum, the MIC remained unaffected at either 5.0 or 7.3 pH (b).	(a) Lamp (1992), (b) Barcia-Macay et al. (2006), (c) Henner and Sitrin (1984), (d) Horká et al. (2014)

For the quinolone class of antibiotics, acidic pH has been shown to reduce antimicrobial activity (Kamberi et al., 1999). This reduction in activity is thought to be caused by ionization of the agents at a lower pH, which results in the inability to penetrate bacterial membranes (Irwin et al., 2013). Ciprofloxacin and levofloxacin have been demonstrated to retain antimicrobial activity within a range of pH 5.4–8.4 and decrease the MIC in increasingly alkaline conditions (Kamberi et al., 1999; Barcia-Macay et al., 2006; Tato et al., 2014).

The antibiotic mode of action can be adversely affected as the environmental conditions change (Figure 9). Aminoglycosides, for example, rely on aerobic bacterial respiration creating an electrical potential for uptake into the cell (Vakulenko and Mobashery, 2003), so an anaerobic environment deep in the biofilm strata reduces their efficacy. With quinolones, it has been shown that, although ciprofloxacin can penetrate *P. aeruginosa* biofilms, it is only active against cells in zones with high pO_2_ and high metabolic activity (Walters et al., 2003).

pH-mediated potentiation of aminoglycosides has been shown to kill bacterial persisters and eradicates *in vivo* biofilms. The application of gentamicin and the clinically compatible basic amino acid L-arginine together affects the sensitivity of bacterial (*S. aureus*, *E. coli,* and *P. aeruginosa*) biofilm persisters to gentamicin in an *in vivo* model as the aminoglycoside efficiency under alkaline conditions increases (Baudoux et al., 2006; Lebeaux et al., 2014). The addition of EDTA, a cation chelator that destabilizes the biofilm matrix, or non-toxic alkaline amino acid L-arginine, which increases the pH of the antibiotic solution in an *in vitro* study, enhanced the activity of gentamicin, amikacin, and vancomycin against biofilms formed by a broad spectrum of bacterial strains responsible for catheter-related bloodstream infections (Lebeaux et al., 2015).

## pH targeted bacterial infection treatment

Implant-associated infections are commonly treated with antibiotics, such as rifampicin, cotrimoxazole, linezolid, clindamycin, minocycline, moxifloxacin, fusidic acid, vancomycin, or daptomycin. However, once biofilms mature, microorganisms in biofilms demonstrate increased tolerance, as discussed earlier. At this stage, device removal surgery is necessary for treatment, which is not always practical since the procedure itself may be prone to complications (Abad and Haleem, 2018; Wi and Patel, 2018). Therefore, it is necessary to develop innovative and effective treatment strategies for bacterial infections, with emphasis on targeted and responsive delivery of therapeutics. New treatment strategies as alternatives to antibiotics include repurposed drugs, antimicrobial peptides and polymers, nucleic acids, small molecules, and bacteriophages (Deusenbery et al., 2021). Another treatment to prevent infection is by modification of materials already used in the medical industry. It is based on modifying the device surfaces so that no bacterial adhesion can occur (Raynor et al., 2009; Treter and Macedo, 2011). Modification of materials that are currently being used by the medical industry is preferred, as they are biocompatible as well as economical. Most of the approaches use materials that are resistant to bacterial adhesion or degradation. Although these strategies are shown to be effective, they are still under development and study (Banerjee et al., 2011; Treter and Macedo, 2011). Therefore, it is essential to study the nature and characteristics of biofilms to achieve a more effective strategy against infections.

Biofilm acidosis can be used not only as a diagnostic reporter but also as a useful feature for targeting therapies to antibiotic-resistant regions. Three principal approaches are used: (1) directly raising pH in the biofilms to mitigate pH-dependent resistance (e.g., with L-arginine, although this may have other biochemical effects too, or increasing local oxygen to encourage aerobic respiration); (2) simultaneously using multiple antibiotics optimized for different microenvironments; and (3) targeting low pH regions near implant coatings or within biofilms for controlled drug release.

**Figure 10:**
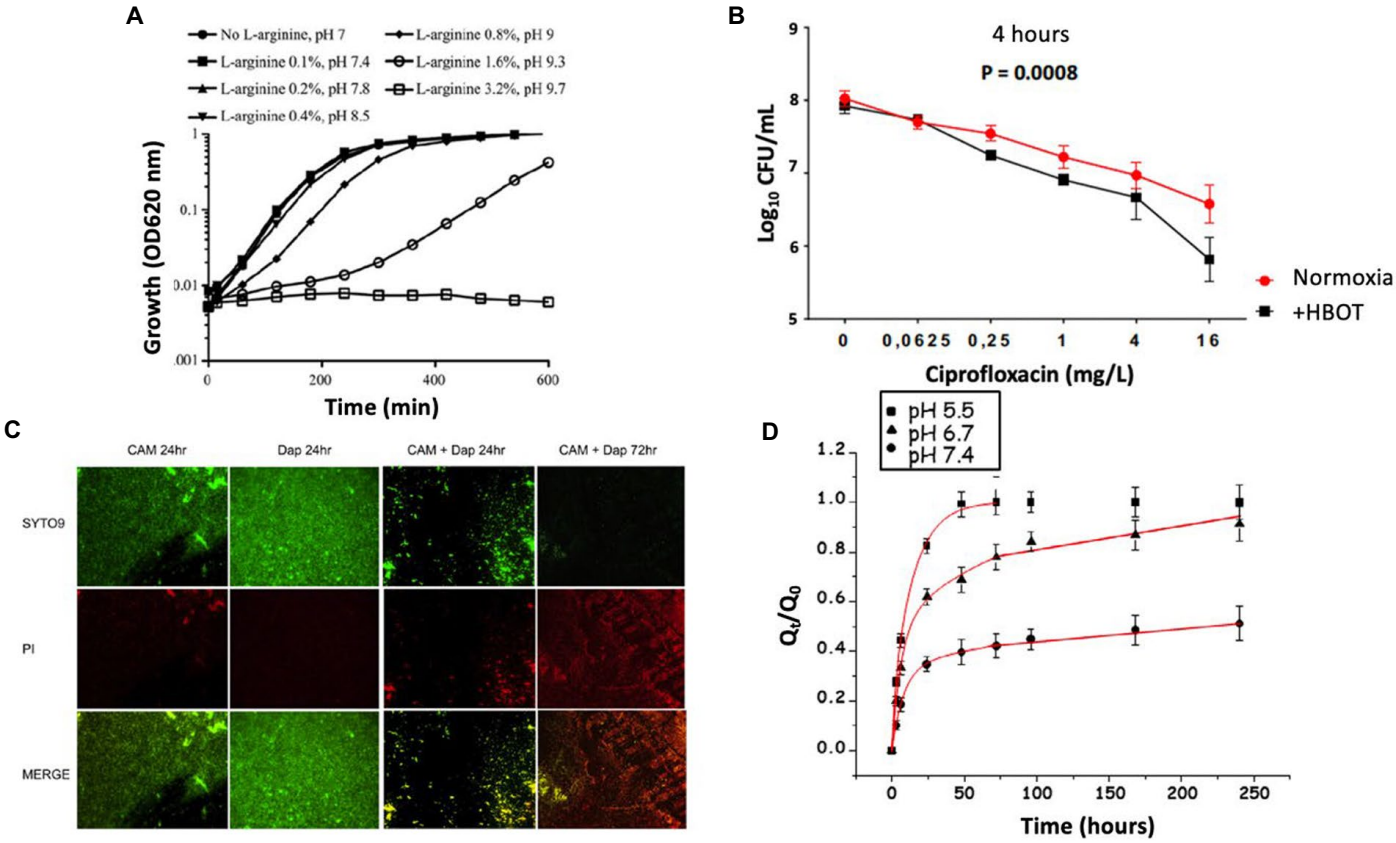
Results of different therapeutic strategies used to target biofilm pH to improve antibiotic activity in biofilms. **(A)** Growth curves in TSB glucose solutions without antibiotics, with increasing concentration of L-arginine for *S. aureus* Xen36 (Lebeaux et al., 2014). **(B)** Bacterial susceptibility to ciprofloxacin (calculated as log_10_ colony forming units/mL) after 4 h HBOT treatment of agarose-embedded *P. aeruginosa* PAO1 biofilms. (Kolpen et al., 2016). **(C)** Fluorescence microscopy images of MRSA on metal surface treated with clarithromycin, daptomycin, and combination of clarithromycin and daptomycin (green indicates live cells and red indicates dead cells; Fujimura et al., 2015). **(D)** The cumulative amount of levofloxacin released (Q_t_) with respect to the initial amount of levofloxacin in the scaffold (Q_0_) versus time at pH 5.5, pH 6.7, and pH 7.4 (Cicuéndez et al., 2018).

In the treatment approach to directly raise the biofilm pH, several studies demonstrate that alkalinization of the medium enhanced the effects of aminoglycosides, resulting in an increase in the antibiotic treatment efficiency (Sabath and Toftegaard, 1974; Moriarty et al., 2007). For example, Lebeaux and co-workers found that raising pH using the harmless basic amino acid L-arginine, resulted in an increase in the killing efficiency of the aminoglycoside gentamicin toward persisters in both *in vitro* and *in vivo* biofilms (Lebeaux et al., 2014). Figure 10A shows the growth curves of luminescent variants of *S. aureus*, *E. coli*, and *P. aeruginosa* (*S. aureus* Xen36) in tryptic soy broth (TSB) glucose solutions, with increasing concentrations of L-arginine without antibiotics (Lebeaux et al., 2014).

Biofilm regions with low oxygen demonstrate antibiotic resistance, partly due to decreased efficiency of oxygen-dependent antibiotics and also due to reduced metabolism (Ciofu et al., 2017). Hyperbaric oxygen therapy (HBOT; 100%, 2.8 bar) has been used to reoxygenate *P. aeruginosa* biofilms, which resulted in an increase in the efficiency of antibiotic treatment (Pakman, 1971; Lima et al., 2015; Kolpen et al., 2016; Jensen et al., 2019). Figure 10B shows that 4 h of HBOT treatment of an oxygen-depleted *P. aeruginosa* biofilm increased the efficiency of the fluoroquinolone, and ciprofloxacin, compared with normoxic conditions (Kolpen et al., 2016). Raising the oxygen levels in the biofilm by HBOT is used as an adjunct or main treatment of infectious diseases, which involves breathing in 100% oxygen at a pressure higher than 1 atm absolute (Çimşit et al., 2009).

Biofilms are differentiated microbial communities with distinct metabolic environments (Povolotsky et al., 2021); therefore, different antimicrobial agents can be combined to target specific regions to prevent or delay resistance (Aaron et al., 2002; Döring et al., 2012). Combination therapy involves using a combination of antimicrobials to treat biofilm-related infections. A combination of ciprofloxacin and colistin (an amphipathic polypeptide) or tetracycline and colistin completely eradicated all *P. aeruginosa* biofilm cells *in vitro* in a study by Pamp et al. (2008). Bactericidal activity of the antimicrobials was based on targeting different metabolic states of the biofilm cells, where colistin killed the biofilm cells with low metabolic activity and ciprofloxacin or tetracycline specifically killed the subpopulation of metabolically active biofilm cells. *In vitro* studies on biofilms formed on metal medical devices showed eradication of staphylococcal biofilms by using clarithromycin with either daptomycin (Fujimura et al., 2015), cefazolin, or vancomycin (Fujimura et al., 2008). Figure 10C shows fluorescence microscope images of methicillin-resistant *S. aureus* (MRSA) on a metal surface stained with a combination of SYTO9 and propidium iodide (PI). The biofilms were treated with antibiotics, clarithromycin, and daptomycin (alone and in combination); clarithromycin is known to have antibiofilm activity, and daptomycin has the ability to readily penetrate biofilms (Fujimura et al., 2015). The results revealed the complete eradication of *S. aureus* biofilms after 72 h of exposure to clarithromycin and daptomycin in combination. Other combinations shown to eradicate MRSA biofilms include ceftaroline and daptomycin (Barber et al., 2015), or vancomycin with fosfomycin (Shi et al., 2014). Therefore, using multiple antimicrobials provide an effective treatment option for eradicating biofilms. Similarly, pH should be one of the considerations due to the pH-dependence of some antibiotics. It is possible to target the pH micro-gradient existing in biofilms by choosing a combination of antibiotics that will work in high and low pH.

A promising approach to treating biofilms is to specifically target the low pH regions for therapy. Similar approaches are used to target acidic regions of tumors. Synthetic polymers that respond to pH have been developed and studied to detect, monitor, or treat infection. Using pH-responsive polymers in drug delivery systems can increase drug efficiency, decrease toxic side effects, facilitate drug absorption and access to the target site, and regulate the drug input with the required timing. To achieve these therapeutic results, polymeric carriers play an important role (Bazban-Shotorbani et al., 2017). Different polysaccharides, such as amylose, guar gum, pectin, chitosan, inulin, cyclodextrin, chondroitin sulfate, dextran, and locust bean gum, have also been used for drug delivery (Reyes-Ortega, 2014). pH-sensitive polymers contain pendant acidic or basic groups that either accept or donate protons in response to the environmental pH. The total number of charged groups on polymer chains determines the overall response of the system to changes in the external pH (Bazban-Shotorbani et al., 2017). In acidic conditions, the polybasic groups are protonated, and the internal charge repulsions between neighboring protonated polybasic groups are increased. These charge repulsions can lead to an expansion in the overall dimensions of the polymer containing the groups (Reyes-Ortega, 2014). At higher pH values, the groups become less ionized, the charge repulsion is reduced, and the polymer-polymer interactions increase, leading to a decreased overall hydrodynamic diameter. These characteristics are used, for example, to obtain pH-responsive hydrogels, which are widely used as carriers in drug delivery systems (Gupta et al., 2002; Rivest et al., 2007).

pH-triggered drug release can be done by incorporating a pH-responsive moiety to the polymer structure, destabilizing a self-assembled polymeric aggregate, or by chemical conjugation of pH-liable linkage between polymers and drugs (Gil and Hudson, 2004; Serra et al., 2006). Cicuéndez et al. (2018) designed pH-sensitive 3D hierarchical meso-macroporous scaffolds with mesopores loaded with the antibacterial agent levofloxacin (Levo). Figure 10D shows the *in vitro* drug release assays from 3D Levo scaffolds at different pH values. The scaffolds demonstrate controlled and pH-dependent Levo release, with increased release at pH relevant to infection (pH 6.7 and 5.5). Pavlukhina and coworkers designed poly (methacrylic acid; PMAA) ultrathin hydrogel coatings that release antimicrobial agents (AmAs) in response to pH variations (Pavlukhina et al., 2010). Gentamicin and an antibacterial cationic peptide L5 were used as AmAs. Adipic acid dihydrazide (AADH) was used as a cross-linker that increases the hydrogel hydrophobicity and provides centers for hydrogen bonding to AmAs. *S. epidermidis* adhesion and colonization were inhibited by releasing the AmAs in response to pH decreases due to bacterial growth. In addition, they could change the antimicrobial release by varying the type of cross-linker (Pavlukhina et al., 2010). In another study, silver nanoparticles triggered by low pH with antibacterial properties were used (Dong et al., 2017). The pH level around the implant surface was 7.4, while during bacterial infection, it dropped to pH 5.5. The pH change was used as a trigger to release silver nanoparticles with antimicrobial properties from the implant surface. Silver has broad-spectrum antimicrobial properties at low concentrations. Silver nanoparticles (AgNPs) are biocompatible with mammalian cells. pH-responsive chitosan nanoparticles from a novel twin-chain anionic amphiphile for controlled and targeted vancomycin delivery (Kalhapure et al., 2017) is another example. The mechanism involved in greater drug release at pH 6.5 could be due to the decrease in ionization of surfactant, a new anionic Gemini surfactant AGS 7, under mildly acidic conditions, resulting in a destabilization of the nanoparticles’ structure. A similar kind of triggered release of methotrexate under acidic conditions was observed for pH-responsive chitosan nanoparticles that were prepared using an anionic surfactant 77 Kl. A summary of the studies on treatment of infection is listed in Table 4.

**Table 4:** Summary of various studies on treatment of infection.

Approach	Treatment technique	Characteristics	Mechanism	References
Directly raising pH in the biofilms to mitigate pH-dependent resistance	Combination of L-arginine and gentamicin against planktonic persisters	Gentamicin supplemented with L-arginine enables eradication of *in vivo* biofilms formed by *S. aureus* and *E. coli via* both a pH-mediated and a pH-independent effect.	L-arginine is basic, which raises the pH resulting in increased efficiency of gentamicin.	Lebeaux et al. (2014)
	Hyperbaric oxygen treatment with 100% O_2_ at 2.8 bar increases bactericidal activity of ciprofloxacin	Enhanced bactericidal activity of ciprofloxacin on oxygen-deficient *P. aeruginosa* biofilms.	Reoxygenation of oxygen-deficient biofilms using hyperbaric oxygen treatment.	Kolpen et al. (2016)
Multiple antibiotics optimized for different microenvironments	Combination of ciprofloxacin and colistin or tetracycline and colistin	Combination of antimicrobials completely eradicated all *P. aeruginosa* biofilm cells *in vitro*.	Bactericidal activity of combination of antimicrobials targeting different metabolic states of cells.	Pamp et al. (2008)
	Combination of clarithromycin and daptomycin	Complete eradication of *S. aureus* biofilms after 72 h of exposure.	Combined antibiofilm activity of clarithromycin and ability of daptomycin to readily penetrate biofilms.	Fujimura et al. (2015)
	Combination of clarithromycin with cefazolin or vancomycin	Complete eradication of *S. aureus* biofilms on titanium medical devices *in vitro*.	Combined antimicrobial activity of clarithromycin with cefazolin or vancomycin.	Fujimura et al. (2008)
Targeting low pH regions for controlled drug release	pH-sensitive 3D hierarchical meso-macroporous (MGHA) nanocomposite	Release of antibacterial agents sustained over time at physiological pH (7.4) and notably increased at infection pH (6.7 and 5.5).	Different protonation state of antibiotic can lead to different interactions between Levo and silanol groups of the mesoporous matrix.	Cicuéndez et al. (2018)
	pH-responsive poly(methacrylic acid) (PMAA) ultrathin hydrogel	Releasing the antimicrobial agents in response to low pH due to bacterial growth.	Increased swelling of hydrogels at pH > 5.5 due to deprotonation and the resulting repulsion between ionized carboxylic acid groups releases the antimicrobial agents.	Pavlukhina et al. (2010)
	pH-dependent release of silver nanoparticles (AgNPs) from titania nanotube arrays (TNT)	Low pH (pH 5.5) trigger release of AgNPs from TNT.	Release of AgNPs which have broad-spectrum antimicrobial properties.	Dong et al. (2017)
	pH-responsive chitosan nanoparticles	Controlled and targeted vancomycin release.	Decrease in ionization of nanoparticles under acidic conditions.	Kalhapure et al. (2017)
	pH-responsive alginate dialdehyde-gentamicin (ADA-Gen) polymer	The acidic environment could trigger the release of ADA-Gen from the multilayer films.	Disruption of the Schiff base bonds of ADA-Gen molecule in an acidic environment.	Tao et al. (2019)
	pH-responsive tobramycin-loaded micelles in nanostructured multilayer-coatings of chitosan/heparin (Tob-loaded CHT/HET)	The coating showed fast release at pH 7.4 and sustained release in acidic conditions.	The polymeric micelles acted as nanovehicles for efficiently loading antibiotic drugs in multilayer coating. Long-term release in acidic conditions with excellent antibacterial activity.	Zhou et al. (2018)
	Polyaniline (PANI) and glycol chitosan (GCS) functionalized persistent luminescent nanoparticles (PLNP@PANI-GCS)	PLNP@PANI-GCS act as pH switchable nanoplatforms for precise photothermal therapy. Low pH causes a change in the charge of the PLNP@PANI-GCS due to the pH-dependent photothermal conversion property of PANI and pH-dependent surface charge transition of GCS.	PLNP@PANI-GCS have a positive charge in acidic microenvironments, interacts with the negatively charged cell wall of bacteria, and activates photothermal effect in acidic microenvironment.	Yan et al. (2020)

## Conclusion and recommendations

Implant-associated infection is a significant problem affecting a large patient population. Conventional oral or systematic antibacterial treatments do not control infections because the drugs cannot penetrate the biofilms and/or are ineffective in dormant or low pH regions. The lack of an effective treatment method to remove the infection, and the bacterial resistance to most therapeutic approaches when they form biofilms on the implant surfaces, highlights the need for more effective and accurate diagnosis methods to detect and monitor implant-related infection. Understanding the biofilm formation process and infection progress can help us to develop and design better and more effective diagnosis and preventive technologies to overcome this problem. In many *in vitro* experiments, single culture biofilms are studied, which makes the experiments easier and capture many essential elements of the growth and physiology, but it should be considered that *in vivo* biofilms are often multi-species. Medical biofilms also have multiple host cell types that influence the local environments.

Low pH is usually associated with infections related to medical implants and can be localized at implant surfaces. The degree of pH drop depends on the type of implant, its location, infection, and patient immune response. The effect of pH on the biofilm depends on the kind of species in the biofilm. The acidic pH of biofilms will affect the antibiotic activity and affect the therapeutic response to a particular drug. This review covered the implant-associated infection process, biofilm formation, and the pH changes during biofilm establishment. The causes and effects of biofilm formation and pH changes were discussed. Different pH measurement techniques showed a pH gradient in the biofilm, which gets more acidic with depth and is heterogeneous throughout the matured biofilm. Regions that have low pH in the biofilm also have low oxygen, which could be a reason for the different effects of heterogeneous regions on compound functions. pH can affect the antibiotic activity and immune cell functions as well as the extracellular matrix. These effects are related to the bacterial species and type of antibiotics. These findings can be used to select the proper antibiotic treatment for a specific biofilm or use of a combination to target different microenvironments. We reviewed *in vitro* studies that used pH changes to detect infection growth. More studies are needed to understand the *in vivo* environment, where additional factors from immune response and antibiotics may affect pH, and low pH from aseptic inflammation may also reduce pH, especially at the early stages of wound healing. While *in vivo* measurements are much more difficult due to optical scattering, attaching sensors directly to the implant surface offers a way forward. In addition to potentially providing measurements for early infection detection and treatment monitoring, elucidating the *in vivo* pH environment will be useful in informing biofilm-specific treatments.

## Author contributions

SB, SK, UW, and CT conducted literature review. SB took the lead in writing the manuscript with support from SK, UW, and CT. JA and T-RT conceived and coordinated the writing of the manuscript. All authors provided critical feedback and contributed to the final manuscript.

## Funding

This study was supported by NIH NIAMS R01 AR070305-01.

## Conflict of interest

The authors declare that the research was conducted in the absence of any commercial or financial relationships that could be construed as a potential conflict of interest.

## Publisher’s note

All claims expressed in this article are solely those of the authors and do not necessarily represent those of their affiliated organizations, or those of the publisher, the editors and the reviewers. Any product that may be evaluated in this article, or claim that may be made by its manufacturer, is not guaranteed or endorsed by the publisher.
